# Discourse, sentiment, and resonance of workplace burnout on Chinese social media: A computational analysis

**DOI:** 10.1371/journal.pone.0346396

**Published:** 2026-08-05

**Authors:** Mengjiao Yin, Yingying Xia

**Affiliations:** 1 School of Business, Wuxi Taihu University, Wuxi, China; 2 AICB laboratory, Wuxi, China; Guangxi Normal University, CHINA

## Abstract

**Background and theoretical context:**

Occupational burnout has evolved from an individual psychological concern into a systemic challenge to organisational sustainability. Traditional burnout research relies predominantly on structured scales or small-scale interviews, making it difficult to capture the spontaneous, socially interactive emotional expressions that employees generate in authentic contexts. Against the backdrop of a discursive shift in Chinese digital workplace culture from individual attribution to structural attribution, social media has become a critical arena in which employees express burnout, seek resonance, and produce collective critical discourse, thereby offering a new perspective on burnout research that goes beyond the scale-based paradigm.

**Research objectives:**

This study aims to systematically examine the discourse structure of contemporary Chinese workplace burnout (how employees talk about burnout, and which themes appear frequently and are invested with meaning) as well as its resonance mechanism (which emotional expressions are more likely to elicit collective online engagement), with a view to revealing the dual structure of workplace burnout discourse.

**Methods:**

Adopting a cross-sectional observational computational social science paradigm with a descriptive and exploratory design, and integrating the theoretical frameworks of emotional labour and affective publics, this study collected texts from five major Chinese social media platforms (Rednote, Bilibili, Weibo, Douyin, and Douban). After data cleaning and line-by-line manual review, 1,276 valid texts were retained. Grounded theory three-layer coding (open coding, axial coding, and selective coding; Fleiss’ Kappa = 0.792) was used to identify discourse themes. A dual-model PCA fusion of RoBERTa and SnowNLP scores was employed to extract sentiment scores, supplemented by human annotation (n = 300; ICC(2,1) = 0.870) for cross-validation. OLS log regression and Poisson pseudo-maximum likelihood estimation (with covariates including follower count, platform, and year) were applied to examine the association between sentiment polarity and like-based engagement.

**Results:**

At the discourse level, grounded coding identified 6 axial themes and 3 core categories. The two highest-frequency themes were Labour Time Deprivation and Intensity Overload (22.7%) and Wage Deprivation and Employment Hardship (21.4%). Workers move beyond individualised narratives of emotional exhaustion, directly naming structural oppression through discourse frames such as Organisational Oppression and Institutional Critique (18.0%) and Passive Resistance and Collective Self-deprecation (16.5%), highlighting a paradigm shift from passive endurance to active critique in contemporary Chinese workplace burnout discourse. At the resonance level, follower count was found to exert a suppression effect on the sentiment-like relationship: after controlling for account exposure, negatively valenced content was significantly associated with higher like counts (PCA: beta = -0.403, p < 0.001), and this direction was fully consistent across three regression model specifications. At the topic level, content pointing to structural oppression (Interpersonal tension: beta = +0.778; Exploitation: beta = +0.656) was significantly more likely to attract online resonance than content focused on individual psychological experience (Sense of defeat: beta = -0.400),

**Conclusions:**

This study demonstrates that contemporary Chinese workplace burnout has evolved from a traditional individual psychological state into a social-affective practice jointly driven by discourse construction and emotional resonance.

## 1. Introduction

Against the backdrop of accelerating global workplace transformation, employee burnout has evolved from an individual psychological concern into a systemic risk to organisational sustainability. According to Mercer’s 2022–2023 Global Talent Trends study, more than 80% of Chinese enterprises have placed burnout mitigation on their priority agenda, viewing it as a core barrier to transformation. Yet despite most employees claiming to be “satisfied” with their current jobs, turnover intentions remain persistently high, exposing a profound disconnect between organisational interventions and employees’ lived experiences [1]. This contradiction suggests that relying on traditional questionnaires or management-imposed frameworks may fail to reach the deep discursive logic and emotional mechanisms that drive burnout. What truly burns employees out remains a question urgently in need of answers grounded in employees’ own narratives.

Employees’ authentic experiences of work-related stress and burnout are increasingly expressed through social media platforms in spontaneous, real-time, and highly contextualised ways. These digital discourses not only constitute an “emotional barometer” of contemporary workplace culture [2, 3], but also, owing to their unstructured nature, high timeliness, and interactive group dynamics, offer researchers a valuable data source that transcends traditional survey methods [4]. Particularly in the Chinese context, when formal communication channels are constrained, anonymous or semi-anonymous online spaces often become the critical arenas in which employees vent emotions, share struggles, and seek resonance [5, 6]. Systematic analysis of such digital narratives therefore holds promise for uncovering the micro-dynamics and collective affective structures of burnout that are obscured by institutional discourses.

Before turning to the specific research questions, it is necessary to briefly outline a diachronic background for readers unfamiliar with contemporary Chinese workplace culture. Around 2010, the mainstream representations of work-related exhaustion in Chinese workplaces broadly followed an individualised attribution model. Employees tended to understand burnout as a personal adaptation problem framed in terms of “insufficient stress tolerance” or “the need to adjust one’s mindset,” a narrative deeply shaped by the ethic of “hardship endurance” and “striving” that has pervaded discourse since the Reform and Opening-Up period [7]. Indeed, scholars have noted that managerial practices in Chinese internet companies frequently couple prolonged overtime with discourses that equate such labour with “loyalty” and “dedication,” thereby disguising structural overwork as employees’ personal choice [8,9]. However, from approximately 2018 onward, marked by the explosive proliferation of indigenous terms including “996,” “内卷” (involution), “躺平” (lying flat), “打工人” (workers), and “社畜” (corporate livestock), Chinese digital workplace discourse underwent a pronounced shift from individual attribution to structural attribution, repositioning workplace pressure as a systemic, institutional predicament that cannot be resolved through personal effort [10,11]. Among these terms, “involution” is particularly significant: anthropologist Xiang Biao has interpreted it as “an endless competitive cycle in which individuals know it to be meaningless yet cannot exit,” a race “that permits neither failure nor withdrawal” [8,12]. In resonance with this, “lying flat” emerged in 2021 as a form of passive resistance to excessive competition and overwork, interpreted by scholars as a nonviolent, non-cooperative refusal by Chinese youth against the “striving ethic” and neoliberal work culture [13]. It is precisely within the context of this diachronic discursive transformation that the question animating this study, namely how employees discuss burnout in digital public spaces, acquires its theoretical weight: it constitutes not merely a measurement of present emotional states, but a capture of an ongoing paradigm shift in discourse.

In light of this, this paper aims to move beyond static definitions or unidimensional measurements of burnout and instead systematically investigates its generative and communicative dynamics through employees’ spontaneous narratives on social media. Specifically, the study addresses two interrelated research questions:

RQ1 (Discourse): How do employees talk about burnout? Which themes appear frequently and are invested with meaning?

RQ2 (Resonance): Which emotional expressions are more likely to elicit engagement and patterns of collective online recognition, thereby forming feedback-reinforced communicative circuits?

To address these questions, this paper constructs an analytical framework integrating natural language processing and social computing. Texts related to workplace burnout were collected from five major Chinese social media platforms. Grounded theory three-layer coding (open coding, axial coding, and selective coding) was applied to allow discourse themes to emerge inductively from the data, with three coders annotating independently and Fleiss’ Kappa used to assess reliability. Dual-model PCA fusion of RoBERTa and SnowNLP was used to extract sentiment scores, supplemented by human annotation (n = 300) for cross-validation. User like counts were used as a proxy for online resonance, and OLS log regression together with Poisson pseudo-maximum likelihood estimation was applied to examine the association between sentiment and topic on engagement, controlling for platform, year, text length, and other confounders. This multi-level analytical pathway enables us to simultaneously capture the semantic content, emotional dynamics, and online circulation patterns of burnout narratives.

This study holds dual significance at both theoretical and practical levels. Theoretically, by integrating discourse analysis, computational sentiment modelling, and resonance mechanisms, we extend burnout research from an “individual symptom” paradigm toward a “social affective practice” framework, offering a new perspective on the collective construction of workplace stress [14]. This approach resonates with Hochschild’s [15] sociological interpretation of emotional labour and further extends Papacharissi’s [16] conceptualisation of affective publics. Practically, the identified high-resonance emotional patterns and key discursive nodes can serve as early warning signals and intervention entry points for organisations, not only revealing whether employees are “exhausted,” but also clarifying why they feel “burned out” and which forms of expression most readily elicit empathy.

## 2. Literature review and theoretical framework

### 2.1 Occupational burnout

Occupational burnout has become an increasingly severe occupational health issue worldwide, not only impairing individual physical and mental well-being but also posing a systemic threat to organisational effectiveness and the quality of public services. The Maslach Burnout Inventory (MBI) defines it as a syndrome comprising three core dimensions: emotional exhaustion, depersonalisation, and reduced personal accomplishment [17]. Distinct from ordinary fatigue or clinical depression, occupational burnout is a work-context-specific phenomenon whose roots lie in structural imbalances within organisational systems rather than insufficient individual psychological resilience.

To explain its underlying mechanisms, multiple theoretical models have been proposed in the literature. Among them, the Job Demands-Resources (JD-R) model is one of the most influential frameworks [18]. This model categorises job characteristics into two types: job demands, such as heavy workloads, time pressure, and emotional burdens, continuously deplete individual energy and directly give rise to emotional exhaustion; job resources, such as autonomy, social support, efficient tools, a sense of control, and a culture of recognition, facilitate goal attainment, buffer stress, and foster engagement. The JD-R model emphasises that occupational burnout becomes a predictable outcome when individuals are chronically exposed to a high-demands, low-resources condition. Yin and Xia [19] extended the application of the JD-R model to freelancers and highlighted individual capital as a third critical factor. Similarly, the Effort-Reward Imbalance (ERI) model, grounded in social exchange theory, posits that burnout risk significantly increases when individuals invest high effort but receive low rewards in terms of compensation, respect, and opportunities for advancement. Additionally, the Demand-Control-Support (DCS) model [20] and Conservation of Resources (COR) theory [21, 22] offer complementary explanations from the perspectives of decision-making autonomy, social support, and resource depletion, respectively.

Empirical studies have shown that occupational burnout is influenced by multiple factors. Job demand-related factors include excessive workload [23,20], time pressure [24], role ambiguity and conflict [25], workplace ostracism [26,27], and technological barriers, such as poorly designed EHR systems that lead to cognitive overload and erode work-life boundaries [18]. In contrast, job resource-related factors, including social support [28], job autonomy, psychological capital (encompassing self-efficacy, hope, optimism, and resilience) [29, 30], emotion regulation capacity [31], and career adaptability [32], have all been empirically validated as effective buffers against burnout risk.

Although existing research has deeply illuminated the theoretical mechanisms, influencing factors, and intervention pathways of occupational burnout, the data sources predominantly rely on structured questionnaires (such as the MBI) or small-scale interviews, making it difficult to capture the spontaneous, dynamic, and socially interactive emotional expressions employees generate in authentic contexts. Particularly in different cultural backgrounds and socioeconomic situations, such as the dramatic transformation of global work patterns in the post-pandemic era, the shift in China’s economic growth trajectory, and the prevalence of “involution” discourse, the experience and articulation of burnout may take on new forms and tensions. The present study is a direct response to this gap. Rather than presupposing the dimensions of burnout, we use computational social science methods to mine employees’ indigenous narratives about work-related exhaustion from large-scale Chinese social media texts, seeking to transcend the universalist assumptions of Western scales and reinterpret the deep social significance of “*what burns you out*?” within the specific context of Chinese digital practice.

### 2.2 Emotional labour

Emotional labour, as a core concept in organisational behaviour, refers to the process by which employees actively regulate and manage their emotions at work to meet organisational or occupational role expectations [33,34]. Since its introduction by Hochschild [15], emotional labour has become a central topic in occupational psychology [35,36,37].

The core of emotional labour lies in individuals’ regulation of emotional expression according to the “display rules” established by organisations or situational contexts [34]. These display rules are enacted through two primary strategies: surface acting refers to employees adjusting only their outward emotional expressions to align with organisational expectations while their inner feelings remain unchanged, essentially constituting emotional pretence [38]. For example, maintaining a smile for customers while feeling distressed internally. This strategy is regarded as high-exhaustion emotional labour because it requires continuous self-monitoring, suppression of genuine emotions, and maintenance of an inconsistent external demeanour, thereby readily giving rise to emotional dissonance and subsequently leading to negative psychological outcomes [37]. Deep acting involves individuals actively reshaping their internal feelings, through cognitive reappraisal or similar mechanisms, to align with the emotions they are expected to express [39]. For instance, genuinely experiencing warmth and friendliness toward customers through positive reframing. Because it reduces the incongruence between inner feelings and outward expression, deep acting is considered a more adaptive emotion regulation strategy [39].

The enactment of emotional labour is influenced by factors at multiple levels. At the individual level, high emotional intelligence helps employees more effectively adopt deep acting strategies [40]; psychological capital, comprising positive psychological resources such as self-efficacy, hope, optimism, and resilience, buffers the negative impact of emotional labour on mental health [41], for example by reducing the risk of workplace deviance [42]; and self-efficacy moderates the effect of emotional labour on well-being [43,44]. At the organisational and contextual level, the intensity of display rules directly determines the level of emotional labour demands [45]; supervisor support mitigates its adverse effects and promotes deep acting [46]; perceived organisational justice influences employees’ modes of emotional investment and their evaluation of work outcomes [47]; work-leisure conflict is positively associated with surface acting and negatively associated with deep acting [48]; technology-mediated communication, in the context of widespread remote work, poses new challenges to establishing emotional connections [49]; and external crisis events, such as the COVID-19 pandemic, significantly intensify emotional labour demands and influence strategy selection through anxiety as a mediator [50,51,52].

Numerous studies have confirmed that emotional labour is associated with occupational burnout, with researchers observing this among journalists [53], sports coaches [54], nurses [55], and office workers [56]. Our study, however, focuses on the broader question of what burns employees out across a wide range of workers, essentially asking: which institutional or cultural emotional rules in contemporary workplaces are being internalised or resisted by employees? Complaints, self-deprecating humour, and accusations on social media constitute reverse expressions of such compulsory emotional labour. Thus, our analysis continues the core concern of emotional labour -- how emotions are appropriated and distorted by the workplace, leading to psychological costs -- but extends the domain from traditional service sectors to digital public spaces. Emotional labour theory will be revisited and extended in the Discussion section in conjunction with the empirical findings.

### 2.3 Affective publics

Affective publics is an important interdisciplinary concept that emphasises the central role of emotion in public communication, social mobilisation, and the construction of collective identity [57]. While traditional public sphere theories regard emotion as a disruptive element to rational discourse, affective publics theory repositions it as a generative mechanism of publicity [58]. Papacharissi [16], as the foundational scholar of this theory, argues that digital media technologies do not merely transmit information but play a crucial role in the soft architectures that filter, channel, and actualise affect. Affective publics are not formed through consensus or information sharing; rather, they connect individuals through the flow and resonance of emotion, generating temporary yet powerful senses of collectivity and identity; for instance, emotional expressions on Twitter have become a key driver for public mobilisation and sustained engagement. Dai [57] further advances this perspective by moving beyond the notion of “networked individualism” and situating affective publics within structural inequalities, treating them as a resource for action.

Affective publics manifest in diverse practical forms in real-world contexts. For example, in digital counterpublics, affect coheres marginalised voices through “stickiness” [59]; in platformed racism, it functions as a tool for reproducing discrimination [60]; in political propaganda, it enhances the persuasiveness of ideologies [61]; and even in urban planning, it interacts with residential density and environmental positivity to influence residents’ well-being [62].

Social media platforms have profoundly reshaped the ecology of affective circulation. Algorithm-driven echo chamber effects continuously reinforce users’ existing emotions and cognitive biases, exacerbating social polarisation and information manipulation [63]. Simultaneously, the rise of networked individualism has shifted social connections from traditional geographically bounded communities toward more dynamic and sparse online relational networks [64], rendering emotion a crucial adhesive for sustaining new forms of publicity. This intertwining of affect and information is especially pronounced in crisis contexts; for instance, online collaborative documents have evolved into risk response mechanisms that integrate emotional expression with factual coordination, revealing the distinctive logic of public affect patterns of engagement in the post-pandemic era [65]. Furthermore, regarding specific public issues, researchers can systematically map public sentiment on social media by integrating computational pipelines that combine topic modelling, clustering, and natural language-based sentiment analysis [66], thereby enhancing the visibility of affective publics research, though caution is warranted against the deep influence of linguistic conventions and regional cultural differences on emotional expression [67]. Such studies have provided key methodological insights for this research.

The resonance dimension that this study focuses on, namely which emotional expressions are more likely to elicit engagement and collective recognition, directly corresponds to the affective publics theoretical framework. We do not merely measure emotional polarity (positive or negative), but rather explore how the intensity of emotion operates as a contagious and motivating force within social networks. For example, a post expressing helplessness that attracts large amounts of responses may mark the emergence of a collectively shared burnout affect. This is precisely how affective publics manifest in workplace issues: burnout is no longer an individual problem, but rather a public emotion that can be perceived, circulated, and empowered.

### 2.4 Theoretical integration and analytical pathway

Although the three theoretical frameworks outlined above originate from different academic traditions, they constitute a coherent analytical chain in the present study, moving from the micro to the macro and from individual experience to collective practice. Occupational burnout theory provides the basic definition and dimensional reference for the research object, enabling us to identify burnout-related semantic structures in social media discourse; emotional labour theory further reveals the emotion regulation and resistance practices of employees under the constraints of organisational display rules, providing theoretical tools for understanding the affective mechanisms behind discourse; and affective publics theory extends the analytical horizon from the individual to the collective, revealing how specific emotional expressions are transformed through digital platforms’ circulation and resonance mechanisms from private experience into resources for public critique.

This three-layer framework corresponds directly to the two research questions of the present study: RQ1 (Discourse) examines how employees talk about burnout, drawing on occupational burnout theory’s conception of burnout’s multidimensionality and emotional labour theory’s analysis of display rules and emotional expression. RQ2 (Resonance) examines which emotional expressions are more likely to elicit collective online engagement, drawing primarily on affective publics theory’s account of emotional fluidity and algorithmically mediated publicity.

The overall theoretical pathway of this study can be summarised as follows: departing from the presupposition of “individual symptoms” in traditional burnout research, passing through emotional labour theory’s sociological reassessment of “emotional practice,” and arriving at the level of patterns of collective online engagement revealed by affective publics theory. This pathway enables us to simultaneously grasp three mutually nested levels of the burnout phenomenon: (1) burnout as individual experience, (2) burnout as emotional labour practice, and (3) burnout as a collective resonance event, thereby providing a unified theoretical foundation for the subsequent discourse-sentiment-resonance analysis.

## 3. Methods

### 3.1 Data sources

The data collection procedure of this study has been approved by the Institutional Review Board of the authors’ affiliated institution (approval number: THU-EC-AICB003), ensuring the legality and privacy protection of online data acquisition and use. Data were sourced from mainstream Chinese social media platforms, including Rednote (n = 810), Bilibili (n = 704), Weibo (n = 703), Douyin (TikTok, n = 773), and Douban (n = 701). Python-based web crawling techniques were used, through calls to each platform’s public API interfaces or through webpage scraping that simulates user behaviour, to enable automated collection of relevant posts, comments, and user updates. Data collection was completed on 15 January 2026, with a time window covering February 2022 to January 2026, in order to capture the dynamic transformation of Chinese workplace discourse in the post-pandemic era [19].

With regard to keyword set construction, a two-stage strategy combining theory-driven and data-driven approaches was adopted. In the first stage, initial seed terms were generated based on the three classic dimensions of the Maslach Burnout Inventory and the main job-demand factors defined by the JD-R model (e.g., “lack of work enthusiasm,” “performance appraisal pressure,” “emotional exhaustion”). In the second stage, two authors independently conducted exploratory searches, incorporating localised expressions such as “996 work schedule,” “corporate livestock,” and “human livestock” into the expanded keyword set, ultimately forming a complete set of 34 keywords (see Supplementary Material S3).

With regard to text selection, inclusion criteria required that texts contain at least one keyword, that content be clearly related to work, occupation, or workplace contexts, that texts be no fewer than 10 Chinese characters in length to ensure semantic completeness, and that publication dates fall within the research time window. Exclusion criteria covered purely promotional content, recruitment information, or marketing material; texts in which keywords appear incidentally but whose context is unrelated to the workplace; posts consisting solely of emoticons or lacking substantive textual content; and repetitive content clearly published in bulk by bot accounts or marketing accounts. Data cleaning adopted a multi-layer filtering mechanism: hash comparison was performed to identify exact duplicates, the SimHash algorithm [68] was applied to filter highly similar content, suspected automated posting accounts were flagged and removed, and texts exhibiting clearly fixed templates or containing substantial promotional language were manually spot-checked and excluded. Following this processing procedure, 3,184 texts were initially obtained.

The selection process then followed a two-stage sequential funnel. At Stage 1, the algorithmic cleaning described above reduced the raw retrieved posts to 3,184 by removing exact duplicates, near-duplicates, suspected bot-generated content, and fixed-template promotional texts. At Stage 2, two members of the research team independently reviewed all 3,184 texts line by line; posts were excluded if they met any of the following criteria: (a) the keyword appeared in a non-occupational context (e.g., fan culture references or entertainment content), (b) the content was semantically ambiguous or too brief to convey a coherent meaning, or (c) the post was clearly unrelated to workplace stress or burnout after reading in full. Disagreements between reviewers were resolved through discussion. This two-stage procedure yielded the final corpus of 1,276 posts retained for analysis. Posts excluded at Stage 2 were not imputed or replaced; their exclusion reflects a data quality boundary rather than missing data in the conventional statistical sense, as the corpus is a purposive rather than a probabilistic sample. Among the 1,276 retained posts, 41 lacked one or more metadata fields required for regression analysis (like count, platform, year, or follower count); these 41 posts were included in the discourse analysis but excluded from the regression models, yielding an analytic sample of N = 1,235 for Section 4.2. The complete dataset is available in Supplementary Material S1.

It should be noted that this study employs purposive sampling rather than probability sampling. The research findings should be understood as a description of the discourse patterns of “users who actively produce discourse in digital public spaces” rather than as a representative estimate of the overall emotional state of Chinese workers. Additionally, the collected social media texts have already been subject to dual filtering by platform content moderation and user self-censorship; the intensity of expression observed in the corpus may systematically understate the authentic emotional intensity that would be observed in unfiltered communication, although this censorship context itself constitutes a generative condition for discourse forms such as ironic institutional critique.

### 3.2 Research design

This study follows a cross-sectional observational computational social science paradigm, adopting a descriptive and exploratory design examining discourse and resonance dimensions. The research questions focus on descriptive exploration and theoretical interpretation, rather than causal inference. Data analysis proceeds at two levels, corresponding to the two research questions.

For RQ1 (Discourse), the three-layer grounded theory coding method [69] was adopted, allowing discourse themes to emerge inductively from the data without any pre-specified framework. The coding procedure was as follows: open coding (extracting initial conceptual labels directly from raw texts), axial coding (identifying logical relationships between concepts and clustering them into higher-order themes), and selective coding (abstracting core categories that integrate the overall framework). Three coders independently conducted axial coding on all 1,276 texts; disagreements were resolved through group discussion; and Fleiss’ Kappa was used to assess inter-rater reliability. Theoretical saturation was determined by the criterion of no new concepts emerging.

For RQ2 (Resonance), like counts were used as a proxy for online resonance. A dual-model cross-validation strategy was adopted to extract continuous sentiment scores and examine their association with resonance. The primary model is a RoBERTa pre-trained language model based on the Transformer architecture (IDEA-CCNL/Erlangshen-RoBERTa-110M-Sentiment), optimised for Chinese tasks and capable of effectively handling informal and ironic expressions in online contexts (J. Zhang et al., 2022) [70]. The auxiliary model is SnowNLP (Naive Bayes), which operates on completely different algorithmic principles and can independently capture sentiment information. After standardising the outputs of both models, principal component analysis (PCA) was performed to extract the first principal component as a composite sentiment score (PC1 explains 61.36% of total variance; both model loadings are 0.707, i.e., equal-weight fusion). The Pearson correlation between the two models was r = 0.227 (p < 0.001), indicating that each captures relatively independent sentiment dimensions. PCA was selected as the fusion method for three reasons. First, it is a data-driven technique that extracts shared variance between two independently derived sentiment signals without imposing a priori weights, reducing the risk of researcher-imposed bias in model combination. Second, as the two constituent models operate on entirely different algorithmic principles and were trained on different corpora, their outputs are only weakly correlated (r = 0.227); the first principal component therefore captures the consensus signal between two sources of measurement while attenuating the idiosyncratic biases of either model alone. Third, the equal loadings of 0.707 on both models confirm that the first principal component represents a genuine equal-weight fusion rather than being dominated by either model, and the 61.36% explained variance indicates that a meaningful proportion of shared sentiment information is retained in a single composite score.

To validate the consistency of PCA composite scores with human judgement, 300 texts were stratified-sampled from the 1,276 texts according to PCA scores, and independently rated by three trained annotators using a 5-point Likert scale. Human validation results showed high inter-rater reliability (Intraclass Correlation Coefficient (ICC(2,1)) = 0.870; Krippendorff’s alpha = 0.935; weighted kappa mean = 0.772), with PCA scores achieving an 85.3% agreement rate with human consensus ratings. Additionally, a detection lexicon of 19 irony marker terms was constructed to conduct a sensitivity analysis on potentially ironic texts (n = 147, 4.7%), assessing the extent to which ironic discourse interferes with sentiment scores.

Informed by Guerrero Contreras et al. [71], who demonstrated that incorporating metadata improves the accuracy of social media sentiment analysis, control variables were added to the models. Ordinary Least Squares (OLS) regression estimates the linear relationship between a continuous dependent variable and one or more predictors by minimising the sum of squared residuals. Because raw like counts are heavily right-skewed (mean = 114.3, median = 2, maximum = 34,639, with 35.1% of observations at zero), a logarithmic transformation was applied to the dependent variable as log(likes + 1), where the constant 1 is added to retain zero-valued observations within the sample. This log-transformed OLS specification models the proportional rather than absolute change in like counts associated with a one-unit change in each predictor. The primary model is formally specified as:


$$\begin{array}{c} \text{log}(likes+\text{1})=\beta_{\text{0}}+\beta_{\text{1}}(sentiment)+\beta_{\text{2}}(platform)+\beta_{\text{3}}(year)+\beta_{\text{4}}(textlength)+\beta_{\text{5}}(keyword)+\beta_{\text{6}}(\text{log}(followers))+\varepsilon \end{array}$$

where β₁ is the coefficient of primary interest. All standard errors are computed using the HC1 heteroskedasticity-consistent estimator to account for non-constant error variance across observations. The primary OLS model is supplemented by two robustness checks: Poisson pseudo-maximum likelihood estimation (PPML, HC1 robust standard errors) and threshold OLS regression restricted to the non-zero-like subsample. All regression models include the following covariates: platform (5 categories, reference = Bilibili), year (2022–2026, reference = 2022), text length (text_len), source keyword (top 10 keywords each coded as a dummy variable, with the remainder grouped as “other,” reference = exploitation), and account follower count (log(followers)). Sentiment scores from RoBERTa, SnowNLP, and PCA were each entered into the regression separately, and results across the three measures were compared to assess the robustness of conclusions.

All analyses were implemented in Python 3.14. Key package versions are as follows: transformers 5.10.1, torch 2.12.0, snownlp 0.12.3, scikit-learn 1.8.0, statsmodels 0.14.6, pandas 3.0.1, and numpy 2.4.3.

## 4. Results

### 4.1 Discourse analysis

Through grounded theory three-layer coding, 29 open codes (see Supplementary Material S2), 6 axial themes (see Table 1), and 3 core categories were identified from the 1,276 texts. Inter-rater reliability assessment showed Fleiss’ Kappa = 0.792 (Substantial agreement), with pairwise Cohen’s Kappa values ranging from 0.704 to 0.842 and a three-coder full agreement rate of 73.1%, meeting the reliability requirements for academic publication [72]. Coding disagreements were concentrated among semantically adjacent themes: A1 (Labour Time Deprivation) and A5 (Organisational Oppression) (some texts simultaneously involved overtime phenomena and institutional critique), A3 (Exhaustion) and A4 (Meaning Dissolution) (anxiety, fatigue, and loss of meaning were intertwined), and A6 (Passive Resistance) and A5 (Organisational Oppression) (the boundary between self-deprecating discourse and institutional critique was blurred). These disagreements were resolved through group discussion. New concepts ceased to emerge at approximately the 1,000th text, indicating that theoretical saturation had been reached.

**Table 1:** Axial coding table (N = 1,276).

Axial code	Constituent open codes (frequency)	Total n	%
A1 Labour Time Deprivation and Intensity Overload	Unpaid overtime (109), Excessive hours and intensity (94), Labour time deprivation and overload (48), Performance appraisal pressure (39)	290	22.7%
A2 Wage Deprivation and Employment Hardship	Wage deprivation and employment hardship (113), Frustrated job-seeking (57), Unemployment and income rupture (57), Wage deprivation (29), Age discrimination (17)	273	21.4%
A5 Organisational Oppression and Institutional Critique	Organisational oppression and institutional critique (153), Institutional exploitation critique (29), Structural critique and class consciousness (23), Workplace interpersonal difficulties (13), Labour rights awareness (10), Management absurdity (6), Supervisory suppression and PUA (6)	230	18.0%
A6 Passive Resistance and Collective Self-deprecation	Passive resistance and collective self-deprecation (70), Passive coping and resistance (47), Collective self-deprecating discourse (40), Sense-of-belonging deficit (22), Craving for respite and self-repair (22), Labour rights awareness (10)	211	16.5%
A3 Physical and Emotional Exhaustion	Emotional breakdown and anxiety (79), Physical and emotional exhaustion (68), Sleep deprivation and health damage (9)	156	12.2%
A4 Meaning Dissolution and Career Disorientation	Meaning dissolution and career disorientation (60), Sense of helplessness and defeat (24), Loss of meaning (23), Career disorientation (8), Despair and giving up (1)	116	9.1%

*Note: Labour rights awareness is simultaneously assigned to A5 and A6, as some texts point toward institutional critique and others toward collective resistance behaviour; frequencies are counted in each corresponding theme and are not double-counted in the total.*

Axial coding clustered the 29 open codes into 6 higher-order themes (see Table 1). A1 Labour Time Deprivation and Intensity Overload (22.7%, n = 290) is the highest-frequency theme, integrating four open codes: unpaid overtime (n = 109), excessive hours and intensity overload (n = 94), labour time deprivation and overload (n = 48), and performance appraisal pressure (n = 39). Expressions such as “unpaid overtime,” “alternating shifts are the norm,” and “if KPI targets are not met you are called in to see the boss” reveal the dual plight of workers: physical exhaustion under institutionalised overwork combined with a deficit in labour rights. A2 Wage Deprivation and Employment Hardship (21.4%, n = 273) focuses on inadequate economic returns and labour market adversity, covering wage deprivation and employment hardship (n = 113), frustrated job-seeking (n = 57), unemployment and income rupture (n = 57), wage deprivation (n = 29), and age discrimination (n = 17). Expressions such as “even at 35 I am constantly rejected,” “can’t find a job,” and “scraping by on next to nothing” portray the multidimensional exclusion that ordinary workers face in the labour market. A5 Organisational Oppression and Institutional Critique (18.0%, n = 230) encompasses critical discourse targeting labour institutions, capital exploitation, and organisational power, ranging from individual complaints such as “squeezing employees to death” to systemic critiques of “following capitalist rules,” as well as specific oppressive forms including supervisory PUA and workplace interpersonal difficulties. A6 Passive Resistance and Collective Self-deprecation (16.5%, n = 211) integrates passive resistance and collective self-deprecation (n = 70), passive coping and resistance (n = 47), collective self-deprecating discourse (n = 40), sense-of-belonging deficit (n = 22), and craving for respite (n = 22). Discourse such as “lying flat,” “slacking off,” and “helping those with workplace anxiety avoid pitfalls” functions simultaneously as emotional venting and as a symbolic practice of collective identity construction and micro-resistance. A3 Physical and Emotional Exhaustion (12.2%, n = 156) is the most direct somatic manifestation of occupational burnout, integrating emotional breakdown and anxiety (n = 79), physical and emotional exhaustion (n = 68), and sleep deprivation (n = 9). Expressions such as “working as an accountant gave me somatic anxiety disorder” and “the night before submitting my resignation I couldn’t sleep” depict the deep erosion of bodily and psychological states by work. A4 Meaning Dissolution and Career Disorientation (9.1%, n = 116), though the lowest in frequency, focuses on the systemic collapse of work meaning and self-worth. From the loss of meaning sense expressed as “I’ve become so muddled I can’t even work out what I’m working for,” to the career disorientation of “at 25, with no goals and no direction,” to the utter abandonment of “there’s no hope at all,” a complete chain of deep internalisation of burnout is presented.

Selective coding distilled three core categories. **C1 Labour Alienation and Structural Oppression (encompassing A1 and A5)** is the external root cause of burnout. Excessive working hours, unpaid overtime, performance squeeze, and the abuse of organisational power together constitute the specific forms of institutional oppression, and workers’ own naming and critique of this oppression (“capitalist exploitation,” “supervisory PUA,” “squeezed to death”) itself constitutes a form of discursive resistance. **C2 Economic Deprivation and Livelihood Crisis (encompassing A2 and A4)** is the deep internalisation pathway of burnout. Starting from external economic pressures such as wage deprivation, failed job-seeking, and unemployment-related income rupture, proceeding through structural blockages such as age discrimination and career ceilings, and ultimately internalising into meaning dissolution, directional loss, and complete abandonment, this forms an internalisation chain of “economic hardship, identity crisis, existential despair.” **C3 Physical and Emotional Exhaustion and Passive Resistance (encompassing A3 and A6)** is the direct consequence of burnout and an agentive response to it. Physical exhaustion, emotional breakdown, and sleep deprivation constitute the somatic manifestations of burnout, while “lying flat,” “slacking off,” and “self-deprecation” are micro-strategies through which workers, under structural constraints, preserve remaining resources and maintain psychological boundaries. The three core categories constitute a progressive theoretical chain: C1 structural oppression simultaneously leads toward C3 physical and emotional exhaustion and C2 economic livelihood crisis, while the deep internalisation of C2 in turn intensifies the passive resistance tendency of C3; the three mutually reinforce each other, jointly constituting the deep logic of contemporary Chinese workplace burnout discourse.

### 4.2 Sentiment and resonance analysis

This study employed a RoBERTa and SnowNLP dual-model cross-validation strategy to extract continuous sentiment scores, fused through PCA into a unified measure, and conducted regression analysis on 1,235 texts with complete metadata (like count, platform, year, text length, and follower count).

The basic statistics of the three sentiment measures are as follows: RoBERTa is overall negatively skewed (mean = −0.146, range [−1.000, 1.000]); SnowNLP is overall near-neutral (mean = 0.009, range [−1.000, 1.000]); the PCA composite score has a mean of 0 by virtue of the standardisation property, with a range of [−1.614, 1.835]. The first principal component of the PCA fusion explains 61.36% of total variance, with both model loadings at 0.707, i.e., equal-weight fusion. Human validation confirmed that PCA scores effectively reflect human subjective perception of textual sentiment (as reported in Section 3.2).

The process of stepwise covariate inclusion revealed a key methodological finding (Table 2). Without controlling for follower count (Models 1–5), the regression coefficient of PCA sentiment scores on like counts was consistently non-significant (p > 0.45) with unstable direction. However, after including log(followers), the coefficients of all three sentiment measures underwent a fundamental transformation, consistently becoming highly significant negative values (RoBERTa: beta = −0.430, p < 0.001; SnowNLP: beta = −0.388, p < 0.001; PCA: beta = −0.403, p < 0.001). This phenomenon is statistically a suppression effect. Follower count operates through two opposing pathways on like counts: the first is an exposure pathway, whereby users with more positive emotional expression tend to accumulate more followers, thereby gaining greater exposure and more likes; the second is a resonance pathway, whereby negatively valenced content more readily elicits the empathy and collective recognition of workers in similar situations, resulting in a higher like conversion rate under equivalent exposure. These two pathways operate in opposing directions and cancel each other out when follower count is not controlled, concealing the true association between sentiment and resonance. After controlling for follower count, the exposure differential is stripped away and the resonance pathway becomes observable.

**Table 2:** PCA sentiment coefficient across stepwise regression models (N = 1,235; dependent variable: log(likes+1); Reference platform = Bilibili; Reference year = 2022; HC1 robust standard errors).

Model	Covariates	PCA coef.	SE	p-value	R²
1	None	−0.039	0.053	0.459	0.000
2	+ Platform	−0.035	0.049	0.470	0.124
3	+ Platform + Year	−0.024	0.047	0.604	0.173
4	+ Platform + Year + Text length	−0.021	0.048	0.659	0.173
5	+ Platform + Year + Text length + Keyword	−0.014	0.048	0.772	0.209
6	+ All above + log(Followers)	−0.403	0.032	<0.001***	0.699

The full model controlling for follower count, platform, year, text length, and source keyword (R2 = 0.699, Adj-R2 = 0.694, F = 159.02, p < 0.001) shows (see Table 3) that a one-unit increase in the PCA sentiment score (i.e., more positive sentiment) is associated with a 0.403-unit decrease in log(likes+1) (beta = −0.403, SE = 0.032, p < 0.001), indicating that, under equivalent exposure conditions, negatively valenced content is significantly associated with higher like counts. The positive association between follower count and like counts is extremely strong (beta = +1.595, SE = 0.040, p < 0.001), confirming that account exposure capacity is the primary driver of like counts. Regarding platform effects, with Bilibili as the reference, Weibo (beta = −0.476, p = 0.001) and Douban (beta = −0.461, p = 0.044) show significantly lower like counts, while Rednote (beta = −0.079, p = 0.571) and Douyin (beta = −0.173, p = 0.165) do not differ significantly, reflecting differences in user interaction mechanisms and content ecosystems across platforms. Regarding year effects, only 2025 shows a marginally significant positive effect (beta = +0.327, p = 0.060); no other years differ significantly from the reference year of 2022, suggesting that the resonance intensity of workplace burnout discourse increased in 2025, though the causal interpretation of this temporal effect requires additional longitudinal data. Regarding topic keywords, topics pointing to structural oppression are significantly associated with higher like counts: “Interpersonal tension” (beta = +0.778, p < 0.001), “Exploitation” (beta = +0.656, p = 0.001), and “Long working hours” (beta = +0.479, p = 0.001) are all significantly above the reference group, while “Sense of defeat” (beta = −0.400, p = 0.002) shows significantly lower like counts. This indicates that topics pointing to external structural oppression are more likely to elicit collective resonance than topics focused on individual psychological experience.

**Table 3:** Full OLS regression model: p-values for Three Sentiment Measures (N = 1,235; Dependent variable: log(likes+1); All models include platform, year, text length, keyword dummies, and log(followers); Reference platform = Bilibili; Reference year = 2022; Reference keyword = exploitation; HC1 robust standard errors).

Variable	RoBERTa p-value	SnowNLP p-value	PCA p-value
Sentiment score	<0.001***	<0.001***	<0.001***
Text length	0.440	0.440	0.440
log(Followers)	<0.001***	<0.001***	<0.001***
Platform: Rednote	0.571		
Platform: Weibo	0.001**		
Platform: Douyin	0.165		
Platform: Douban	0.044*		
Year 2023	0.317		
Year 2024	0.455		
Year 2025	0.060.		
Year 2026	0.532		
Keyword: Interpersonal tension	<0.001***		
Keyword: Exploitation	0.001**		
Keyword: Long working hours	0.001**		
Keyword: Sense of defeat	0.002**		
Keyword: Lack of belonging	0.275		
Keyword: Overtime	0.393		
Keyword: Performance pressure	0.768		
Keyword: Lack of work enthusiasm	0.535		
R2	0.699	n/a	0.699
Adj-R2	0.694	n/a	0.694

Note: Platform and year p-values are identical across the three sentiment columns, as all covariates other than the sentiment measure are held constant. *** p < 0.001, ** p < 0.01, * p < 0.05,. p < 0.1

To assess whether the primary finding, namely the negative association between sentiment positivity and like counts after controlling for account follower exposure, is sensitive to modelling assumptions, three additional specifications were estimated alongside the primary OLS model (see Table 4). In the Poisson pseudo-maximum likelihood estimation (PPML), the PCA coefficient was −0.590 (SE = 0.092, p < 0.001), with Pseudo-R2 = 0.820. In the threshold OLS regression (excluding 386 zero-like texts, n = 849), the PCA coefficient was −121.916 (SE = 51.249, p = 0.017). In the primary OLS log regression, the PCA coefficient was −0.403 (p < 0.001). The PCA coefficient is significantly negative across all three model specifications, with a fully consistent direction, indicating that the conclusion that “under equivalent exposure conditions, negatively valenced content is significantly associated with higher like counts” is robust across model specifications.

**Table 4:** Robustness checks: PCA Sentiment Coefficient Across Three Regression Model Specifications (All models include platform, year, text length, keyword dummies, and log(followers)).

Model	Dependent variable	PCA coef.	SE	p-value	N	Notes
OLS log(likes+1)	log(likes+1)	−0.403	0.032	<0.001***	1,235	Primary model, HC1
Poisson PPML	Raw like count	−0.590	0.092	<0.001***	1,235	HC1; Pseudo-R2 = 0.820
Threshold OLS (likes>0)	Raw like count	−121.916	51.249	0.017*	849	386 zero-like obs. excluded

Note: The dependent variable in the threshold OLS model is raw like count (without log transformation); coefficients are not directly comparable in magnitude with those of the primary OLS model, and are presented solely to verify directional consistency. * p < 0.05, *** p < 0.001.

Taken together, the three specifications confirm that the direction and significance of the sentiment-resonance association is not an artefact of the log transformation, the treatment of zero-like observations, or the choice of distributional assumption, but represents a consistent pattern across modelling frameworks.

## 5. Discussion and conclusions

### 5.1 Discourse structure and meaning construction of workplace burnout (RQ1)

The grounded theory coding of this study reveals the deep structure of contemporary Chinese workplace burnout discourse. This structure exhibits significant contextual differences from mainstream Western burnout research that are worth examining in depth. The Maslach Burnout Inventory defines occupational burnout as comprising three dimensions: emotional exhaustion, depersonalisation, and reduced personal accomplishment [17]; this framework presupposes that burnout is primarily an individual-level psychological state. However, this study finds that burnout discourse on Chinese social media is not centred on individual psychological symptoms, but is deeply embedded in a narrative framework of structural critique (C1). The highest-frequency themes, A1 (Labour Time Deprivation and Intensity Overload) and A5 (Organisational Oppression and Institutional Critique), both point to systemic issues in labour institutions and capital relations, with terms such as “exploitation,” “capitalists,” “slaves,” and “class” appearing frequently in the corpus. This stands in direct dialogue with the work of Y. Yang and Jiao [7] on how managers in Chinese internet companies discursively frame excessive overtime as “loyalty” and “dedication”: this study suggests that workers in digital public spaces appear to be engaging in a systematic deconstruction of this discursive logic, reframing institutionally imposed “personal choice” as “structural oppression.” J. J. Wang [8] has likewise documented how the management culture of Chinese internet companies institutionalises the “996” work regime through cultural and control mechanisms; this study’s discourse data corroborates and extends that critique from a bottom-up perspective, suggesting that workers not only perceive oppression but may have developed a discourse system for naming and circulating it. This discursive shift from individual attribution to structural attribution is highly consistent with M. Liu and Chen’s [10] critical discourse analysis of the “996” discourse. They found that, from around 2018, Chinese digital workplace discourse underwent a paradigm shift from “pressure is a personal adaptation problem” to “pressure is an institutional predicament,” with indigenous terms such as “involution,” “lying flat,” and “human livestock” as the linguistic vehicles of this shift [11,8]. The present study provides empirical support for this observation through large-scale corpus analysis and further reveals the specific distributional structure of these discourses across six thematic dimensions.

The relatively high frequency of A6 is particularly noteworthy. Online discourses such as “human livestock,” “corporate livestock,” “slacking off,” and “lying flat” are not only outlets for individual emotional venting, but also symbolic practices of collective identity construction. From the perspective of emotional labour theory [15], this type of discourse constitutes a distinctive “performance of non-performance”: through strategies of non-response, non-participation, and emotional withdrawal, workers actively sever the organisational chain of emotional resource appropriation. This differs both from surface acting’s adjustment of outward expressions and from deep acting’s reshaping of inner feelings [34], and instead constitutes a direct refusal to bear the moral responsibility of emotional labour. Hsu’s [14] research on “lying flat” discourse demonstrates that this refusal is not simply passive idleness, but a nonviolent, non-cooperative resistance to the “striving ethic” and neoliberal work culture; Lin and Gullotta [13] hold a similar view. The findings of this study further suggest that this resistance may have consolidated into a stable discursive form and obtained widespread group resonance across social media platforms.

A4, while relatively low in frequency, carries theoretical significance that cannot be overlooked. This theme corresponds to the state of work resource depletion described by the JD-R model [18]: when individuals are chronically exposed to a high-demands, low-resources structural predicament, the systemic collapse of work meaning and personal accomplishment is theoretically expected as a predictable outcome [18]. Maunz and Glaser’s [22] longitudinal study likewise confirms that sustained declines in psychological need satisfaction directly lead to the loss of work meaning and subsequently to burnout. In the corpus of the present study, expressions of complete abandonment such as “just going through the motions,” “it’s just a way to make a living,” and “I’ve resigned myself to it” perhaps reveal a terminal state of this pathway: burnout is no longer exhaustion, but an existential abandonment of the entire system of work meaning.

Regarding the theoretical relationships among the three core categories, C1 Labour Alienation and Structural Oppression is interpreted as the institutional root cause of burnout, C2 Economic Deprivation and Livelihood Crisis as its deep internalisation pathway, and C3 Physical and Emotional Exhaustion and Passive Resistance as burnout’s direct consequence and agentive response. One possible theoretical explanation, drawing on Conservation of Resources (COR) theory, is that when workers’ core resources...the spiral depletion of resources may on the one hand contribute to identity crisis and existential despair through pathways such as frustrated job-seeking and meaning dissolution (C2), and on the other hand be associated with physical exhaustion and emotional breakdown, as well as micro-resistance practices such as slacking off and lying flat (C3) [21,22]. This chain resonates with Zheng and Qiu’s [9] analysis of how hegemonic despotism sustains long working hours in Chinese internet firms, and provides supplementary evidence from the perspective of discourse practice.

### 5.2 Association between sentiment polarity and online resonance (RQ2)

This study finds that, after controlling for account follower count, platform, year, and other confounding variables, negatively valenced content is significantly associated with higher like counts. This appears on the surface to contradict the intuition that “positive content spreads more widely,” but from the theoretical perspective of affective publics and collective identity, it suggests a plausible internal logic.

Papacharissi’s [16] affective publics theory holds that digital publicity is not driven by rational consensus, but is woven through the flow and resonance of emotion; individuals form temporary yet powerful collective identifications on algorithmically mediated platforms by sharing anger, a sense of absurdity, or ironic humour. The resonance data of this study provide empirical support for this theoretical claim: in the context of Chinese workplace burnout discourse, a post describing “being exploited,” “overtime without pay,” or “unable to find a job” is not merely information for workers in similar situations, but an existential confirmation. A “like” in this context is not merely an interactive behaviour, but a low-cost gesture of collective solidarity. Dai [57] further notes that affective publics do not merely remain at the level of “networked individualism” but are embedded in structural inequalities, constituting a resource for action. The finding of this study that topics pointing to structural oppression obtain more likes than topics focused on individual psychological experience precisely corroborates this judgement: a narrative framework that translates individual suffering into structural critique appears more likely to break through the boundaries of the private sphere and enter the domain of collective resonance.

This finding is consistent with the implicit logic in emotional labour theory that “suppressed emotions have higher infectiousness” [33,15]. When content corresponding to the A5 Supervisory Suppression and PUA theme circulates publicly on platforms, what may be activated in viewers is not only sympathy for the individual situation described, but a mirror-like confirmation of their own similar experiences, suggesting that a “like” in this context may function as a miniature “collective unloading of emotional labour.” Liu and Guan’s [5] research on informal organisational communication channels in the Chinese context likewise points out that when formal communication channels are constrained, anonymous or semi-anonymous online spaces become critical loci for emotional venting and resonance construction, providing an institutional explanation for the platform resonance mechanisms observed in this study.

The finding of a suppression effect for follower count carries important methodological implications. When follower count is not controlled, the emotional effect is entirely concealed, indicating that account exposure capacity is an indispensable confounding variable in social media research. Fernandez- Gavilanes et al. [67] have already noted the deep influence of linguistic conventions and regional cultural differences on emotional expression in sentiment analysis research; this study further reveals the suppression mechanism of account follower scale on the sentiment-engagement relationship, providing a concrete methodological warning for subsequent social media sentiment research: failing to distinguish between “exposure volume” and “resonance conversion rate per unit of exposure” will systematically misestimate the relationship between sentiment polarity and online engagement.

Furthermore, the irony sensitivity analysis warrants elaboration here. All three sentiment measures showed a higher rate of positive classification among ironic texts (59.9% to 70.1%) compared to non-ironic texts (35.6% to 47.3%), indicating that critical discourse constructed from irony markers such as “haha,” “blessing,” and “great” may be misclassified as positive sentiment by sentiment models, thereby to some extent underestimating the overall intensity of negative sentiment in the corpus. In the social media context of workplace burnout, irony is precisely one of the important rhetorical strategies through which workers express critique. Future research should develop dedicated irony detection models for the Chinese workplace context, or adopt mixed methods such as human annotation supplemented by model inference, to improve the accuracy of sentiment analysis in this type of discourse. This limitation has been noted in Section 5.3.

The finding that 2025 like counts are significantly higher than those of the reference year is consistent with the background of accumulating labour market pressures. Yin and Xia [19] have likewise noted that the post-pandemic economic downturn may give rise to burnout experiences and modes of expression that differ from those of the pre-pandemic period; the temporal effect of this study provides discourse-level corroboration for that inference, though the causal mechanisms behind it await further examination with longitudinal data.

### 5.3 Practical implications and limitations

The high-resonance discourse themes and emotional patterns identified in this study can provide organisational managers with an early-warning perspective that goes beyond satisfaction surveys. When structurally critical topics such as “exploitation,” “interpersonal tension,” and “unpaid overtime” appear consistently at high frequency in employees’ social media discussions and elicit large numbers of likes, managers should treat this as a signal of systemic organisational problems rather than isolated individual emotional outbursts, thereby shifting the intervention focus from individual psychological counselling toward institutional improvement, and prioritising structural issues such as working hours, pay equity, and organisational power relations.

This study also has the following limitations that are worth acknowledging. First, regarding the platform-mediated nature and representativeness constraints of the sample: the corpus originates from five Chinese social media platforms, and all texts have already been subject to dual filtering by platform content moderation and user self-censorship. Schmidt et al. [4] note that social media data has the advantage of high timeliness and interactivity as a research source, but is also deeply shaped by platform logic; in the context of Chinese internet governance, direct critique of labour institutions may face visibility restrictions and the most intense expressions may already have been screened out by algorithmic or human review. B. Zheng and Davison [6] likewise note that Chinese employees’ discourse practices on social media are subject to the dual constraints of mixed public-private contexts and relationship-maintenance logic. The findings of this study should therefore be understood as a description of the discourse patterns of “users who actively produce discourse in digital public spaces,” rather than as a representative estimate of the overall emotional state of Chinese workers; this boundary has been similarly articulated in the discourse research of Y. Yang and Jiao [7]. Second, regarding the limitation of like counts as a proxy for resonance: like behaviour is influenced by multiple factors including platform algorithmic recommendation, posting time, and account type. Amendola et al. [63] have shown that algorithm-driven echo chamber effects continuously reinforce users’ existing emotions and cognitive biases, meaning that the sentiment-like associations observed in this study may partly reflect the differential algorithmic recommendation of negatively valenced content rather than purely user resonance choices. Additionally, likes can only capture explicit positive engagement and cannot reflect emotional divergences in comments, critical recontextualisation in reposts, or the emotional states of silent audiences. Future research may incorporate comment sentiment analysis [66] and repost network analysis to more completely characterise the multidimensional structure of online resonance. Third, regarding the causal limitations of the cross-sectional design: this study adopts a cross-sectional, descriptive, and exploratory design, and none of the associational findings support causal inference. Future research should introduce longitudinal tracking or quasi-experimental designs to provide causal verification. In particular, the mechanisms underlying the association between negative sentiment and higher like counts, including emotional contagion, collective solidarity, and algorithmic preference, need to be identified through experimental designs. Fourth, the irony sensitivity analysis suggests that some critical discourse may be misclassified as positive sentiment, thereby to some extent underestimating the overall negative sentiment intensity. Future research should develop dedicated irony detection models for the Chinese workplace context to improve sentiment analysis accuracy.

## Supporting information

S1 File https://osf.io/3ju8d
(CSV)

S2 File https://osf.io/2jqm6
(MD)

S3 File https://osf.io/zytwc
(MD)
