# Supplementary material for: Discourse, sentiment, and resonance of workplace burnout on Chinese social media: A computational analysis
Source: PLoS One. 2026 Aug 5;21(8):e0346396. doi: 10.1371/journal.pone.0346396 (PMC13440837; doi:10.1371/journal.pone.0346396)
Supplement: Supplementary file 1 [file pone.0346396.s004.docx]

**General Response to All Reviewers**

Before addressing the specific comments, we would like to express our deepest gratitude to the Editor and all reviewers for taking the time to evaluate our manuscript and for providing constructive suggestions that have substantially improved the paper. We also wish to clarify an issue raised by Reviewer #1 regarding the AI-generated content rate. We sincerely apologize for any concern this may have caused. As the authors' native language is Chinese, our command of English may not meet the standards required for professional academic writing. We believe that expressing our ideas in informal, error-prone English would diminish the reading experience for specialist audiences. For this reason, we used AI to translate the original Chinese manuscript (including this response letter), which accounts for the elevated AI detection rate. We wish to assure the Editor and reviewers that every sentence in the Chinese manuscript was written and originated entirely by the authors; if required, we are happy to provide the original Chinese draft. Furthermore, every sentence of the translated version was read and reviewed by the authors to ensure that the intended meaning was faithfully conveyed. We therefore respectfully invite the Editor and reviewers to exercise informed judgment: a mechanical AI detection rate cannot serve as a measure of academic integrity; what matters is whether the core ideas originate from the human authors and whether the research data are authentic.

Regarding the order of revision, we first followed the suggestions of Reviewer #3, whose comments were the most critical, and addressed all issues point by point. We then incorporated the suggestions of Reviewer #2, whose comments were more measured, as supplementary revisions. Finally, we checked whether all revisions were consistent with the overarching and structural guidance provided by Reviewer #1. We accordingly recommend that reviewers read the responses in this order.

**Reviewer #1:**

*1) Rewrite the abstract to include a brief description of the problem/theory, objectives, methods, key results, and conclusions.*

We sincerely thank the reviewer for this suggestion regarding the structural completeness of the abstract. We fully agree that the original abstract was structurally deficient: too much space was devoted to describing findings and theoretical interpretations, while the statement of the research problem, the definition of research objectives, and the summary of research methods were relatively underdeveloped, failing to present the five key elements identified by the reviewer (problem/theoretical background, objectives, methods, key findings, conclusions). This structural omission made it difficult for readers to form a clear sense of the study's overall positioning before engaging with the main text. We sincerely apologize for this and have substantially rewritten the abstract in accordance with the reviewer's suggestion. The revised abstract is organized according to the standard structure of "Problem/Theoretical Background — Objectives — Methods — Key Findings — Conclusions."

*2) The introduction should include a brief, solid theoretical basis and justification.*

We sincerely thank the reviewer for this suggestion regarding the theoretical grounding of the introduction. We fully agree that the introduction should, within as concise a space as possible, provide a solid theoretical anchor and justification for the subsequent analysis, enabling readers to form a clear sense of the study's theoretical positioning before encountering the specific research questions. We wish to respectfully note that this comment is internally connected to two concerns raised by another reviewer: "the integrative logic among the three theoretical frameworks of occupational burnout, emotional labor, and affective publics should be articulated more clearly" and "international readers lack the necessary diachronic background on contemporary Chinese workplace culture." To address these interconnected concerns, we have made the following substantive additions to the introduction and literature review sections of the revised manuscript, which simultaneously constitute a direct response to the reviewer's present comment:

First, we have added a concise diachronic cultural background paragraph following the second paragraph of the introduction. This paragraph provides readers with an account of the transformation in Chinese workplace discourse since the 2010s from "individual attribution" to "structural attribution," and in particular explains the semantic evolution of indigenous terms such as "involution (neijuan)," "lying flat (tangping)," and "internal friction (neihao)" along with their scholarly interpretations (e.g., anthropologist Xiang Biao's classic definition of "involution"). This addition provides a solid empirical anchor for the study's theoretical justification, establishing that what this study concerns is not an abstract psychological question but an observable paradigm shift in Chinese workplace discourse.

Second, we have added Section 2.4, "Theoretical Integration and Analytical Approach," to the literature review. This section explicitly articulates the functional differentiation and interrelationship among the three theoretical frameworks — occupational burnout, emotional labor, and affective publics — within the present study: burnout theory provides the basic definition of the research object; emotional labor theory furnishes explanatory tools at the level of individual experience; and affective publics theory furnishes explanatory tools at the collective level. Together, they constitute a coherent analytical chain moving from "individual symptoms" through "affective practice" to "collective resonance," and correspond one-to-one with the study's three research questions. This integrative account enables the research questions posed at the end of the introduction to be anchored within a unified theoretical framework rather than suspended among three independent theoretical fragments.

*3) The methods and materials section should include the type and design of the research, unit of analysis, how the sample was selected, eligibility criteria, variables analyzed, data collection period, data extraction procedures, and the methods used for the analysis of quantitative and qualitative data. This section must be clear and precise to ensure reproducibility.*

We sincerely thank the reviewer for these detailed suggestions regarding the completeness and reproducibility of the methods section. We fully agree that the methods and materials section should systematically present the research type and design, unit of analysis, sample selection, inclusion criteria, variable definitions, data collection period, data extraction procedures, and analytical methods, in order to ensure reproducibility. This comment is closely aligned with another reviewer's suggestion that "more detailed information should be provided on the keyword selection process, inclusion/exclusion criteria, handling of duplicate and automated content, sampling logic, and validation of the automated classification procedure." To systematically address these interconnected concerns, we have made substantive expansions to Section 3 of the revised manuscript, covering all the elements listed in the reviewer's comment.

Regarding research type and design: we have added a clear statement at the beginning of Section 3.1 of the revised manuscript, positioning this study as a computational social science study based on digital trace data from social media, with a cross-sectional descriptive and exploratory design. The research questions focus on descriptive exploration and theoretical interpretation of the three dimensions of "discourse — sentiment — resonance," rather than causal inference.

Regarding the unit of analysis: Section 3.1 of the revised manuscript explicitly states that the basic unit of analysis is the individual social media post/comment, rather than user accounts or topic aggregates. Each text enters the analysis as an independent observation unit in the subsequent topic coding, sentiment scoring, and engagement recording, maintaining a clear correspondence between the sample size (n = 3,691) and the statistical models (ZINB and threshold regression).

Regarding sample selection and inclusion/exclusion criteria: we have added detailed operational information to Section 3.1 covering two aspects. First, sampling adopts purposive sampling rather than probability sampling, conducting systematic searches across five major Chinese social media platforms (Xiaohongshu, Bilibili, Weibo, Douyin, and Douban) using 34 keywords constructed through a two-stage "theory-driven plus data-driven" strategy; the complete keyword list is available in the open-access repository. Second, we explicitly list inclusion criteria (containing at least one keyword, clearly related to work/occupational/workplace contexts, at least 10 Chinese characters in length, published within the research time window) and exclusion criteria (purely promotional or recruitment content, texts where keywords appear out of workplace context, posts consisting solely of emoticons, content mass-published by bot or marketing accounts), and describe the specific procedures for handling duplicate and automated content through a multi-layer filtering mechanism (hash comparison, SimHash approximate deduplication, account-level filtering, content pattern filtering).

Regarding variable definitions: the revised manuscript explicitly identifies three categories of core variables. (1) Discourse variables: thematic labels (label, integer values 0–10) determined jointly through BERTopic clustering and LLM coding. (2) Sentiment variables: sentiment scores (senti, continuous variable ranging from −1.000 to 0.999) output by the RoBERTa model. (3) Resonance variables: raw like counts (likes) and their log-transformed values (log(likes + 1)). For the temporal and geographic analyses, IP-registered location and posting timestamps are used as auxiliary variables.

Regarding the data collection period and data extraction procedures: Section 3.1 of the revised manuscript explicitly states that the data collection window spans February 2022 to January 2026 (covering approximately four years of the post-pandemic era), with data gathered using Python-based web crawling techniques through calls to publicly available platform APIs or by simulating user behavior to scrape web pages. A epistemological declaration on the data production process has been added at the end of Section 3.1, acknowledging that the collected texts are discursive products filtered through the dual mechanisms of platform content moderation and user self-censorship — a statement that also responds to the reviewer's concern regarding "the potential distorting effect of censorship on the data."

Regarding data analysis methods: Section 3.2 of the revised manuscript fully presents the methodological approach for each of the three research questions. For RQ1 (discourse): BERTopic topic modeling is used (with complete parameter configurations for UMAP dimensionality reduction and HDBSCAN clustering), and the Qwen-Plus large language model classifies all texts into thematic categories based on pre-established topic definitions; a three-step validation procedure (stratified sampling, independent double-coding, three-way inter-rater agreement: Fleiss's Kappa = 0.782) ensures coding reliability. For RQ2 (sentiment): a Chinese-optimized RoBERTa pretrained model is used to extract sentiment scores, with reliability assessed through a two-level reliability check (overall random verification and targeted irony verification, with a positive-sentiment classification accuracy of 94.67%). For RQ3 (resonance): Zero-Inflated Negative Binomial (ZINB) regression is used to model the sentiment–engagement relationship, with threshold regression as a robustness check; a bivariate ZINB model incorporating thematic category as a categorical independent variable further examines heterogeneous effects at the thematic level. All analysis code, keyword lists, system prompts, and reliability verification materials have been uploaded to the open-access repository to enable full reproducibility.

Regarding qualitative data analysis methods: we wish to clarify to the reviewer that this study does not employ qualitative methods; it is a purely computational analysis study. During the revision process, we carefully considered supplementing the existing interpretations with in-depth interviews with original posters to provide qualitative triangulation, but after careful deliberation decided not to include such a supplement in the present manuscript. The reason is that a qualitative supplement of insufficient scale (e.g., 8–10 interviews) would not support rigorous methodological triangulation and might instead undermine the study's methodological integrity by appearing perfunctory; a mixed-methods study of adequate scale would require months of additional fieldwork, exceeding what can reasonably be undertaken within a single revision. Based on this consideration, we have chosen the more honest approach of clearly delineating the epistemological boundaries of this study as a purely computational analysis, and explicitly designating mixed-methods supplementation as a direction for future research.

*4) Regarding ethical considerations, there is no mention of whether the Ethics Committee that approved the research protocol issued an exemption from the requirement for informed consent, given that publicly available data from the internet was used; nor is there any mention of which data protection policy was applied.*

We sincerely thank the reviewer for the rigorous attention to research ethics. We fully agree that research based on publicly available social media data, while not involving direct interaction with human participants, must nonetheless provide explicit statements regarding both the waiver of informed consent and the data protection policy applied.

The data collection and analysis procedures in this study have received formal approval from the Institutional Review Board of the authors' affiliated institution (Approval No. THU-EC-AICB003). Given that all data used in this study originate from texts publicly posted by users on open social media platforms without access restrictions, the IRB approved a waiver of informed consent in accordance with the Declaration of Helsinki and relevant Chinese regulations governing human subjects research. This waiver is based on three considerations: first, the study involves no direct contact with, intervention upon, or experimental manipulation of users, and consists solely of passive observational collection of publicly available digital trace data; second, users who actively publish content on public platforms have a reasonable expectation of the public visibility of their posts; third, the research design does not involve the individual identification or profiling of any specific user, with all analyses conducted at the aggregate level (thematic, sentiment, geographic, and temporal distributions).

Regarding data protection policy, we have strictly adhered to the core principles of data minimization and user de-identification. Specifically: (1) at the data collection stage, we retained only fields directly relevant to the research questions — text content, posting timestamp, IP-registered location (provincial level), and like count — while deliberately forgoing collection of usernames, profile pictures, personal homepage links, detailed geographic locations, device information, or any other sensitive fields that could be used to identify individuals; (2) at the data storage stage, any personally identifiable information that may appear in raw texts, such as usernames and @mentions, was automatically cleaned or anonymized during preprocessing; (3) at the data release stage, the dataset uploaded to the open-access repository has been further de-identified, retaining only aggregate-level variables including text content, topic label, sentiment score, provincial IP-registered location, and log-transformed engagement volume, with no information traceable to individual users; (4) regarding data retention, the original collected data are used solely for the analytical purposes of this study and will be stored securely in accordance with IRB requirements upon completion of the research.

*5) Specify the following: how methodological triangulation was performed between qualitative, computational, and theoretical data. Transparency in data preprocessing. How the validity of sentiment measurement and analytical coherence was determined, ensuring the reliability of the results and the study's reproducibility.*

We sincerely thank the reviewer for these detailed concerns regarding methodological rigor. This comment encompasses three interrelated technical requirements: methodological triangulation (integration among qualitative, computational, and theoretical data), transparency in data preprocessing, and validity assessment of sentiment measurement and analytical coherence.

We first wish to clarify an important point regarding the study's methodological positioning: this study does not employ qualitative data (such as interviews, focus groups, or field observations); it is a purely computational social science study whose data derive entirely from social media texts as a single type of digital trace data. Although this study does not include triangulation in the narrow sense of "qualitative–computational" integration, we have implemented multi-level internal methodological triangulation in the revised manuscript, comprising: (1) triangulation at the discourse level — a consistency check among BERTopic's statistical clustering output, LLM classification based on topic definitions, and independent manual verification by two authors (Fleiss's Kappa = 0.7827), constituting a direct operationalization of "computational–human" agreement; (2) triangulation between method and theory — we established a bidirectional mapping between the indigenous discourse topics identified by BERTopic (such as "Internal Friction Resistance" and "Institutional Absurdity") and theoretical concepts drawn from emotional labor theory (such as "display rules" and "emotional dissonance") and affective publics theory (such as "affective flows"), following the in vivo coding principle (i.e., respecting the indigenous conceptual systems already employed by the research subjects themselves); (3) statistical robustness triangulation — for the resonance analysis addressing RQ3, we simultaneously employed ZINB regression and threshold regression as cross-validation, with convergence in core conclusions across both models providing mutual methodological support for the robustness of the findings.

We have made systematic expansions to Section 3.1 of the revised manuscript, explicitly describing each critical step of the data preprocessing procedure: (1) keyword selection adopted a two-stage "theory-driven plus data-driven" strategy; (2) inclusion and exclusion criteria are explicitly listed; (3) deduplication and anti-automation filtering employ a four-layer mechanism (hash comparison, SimHash approximate deduplication, account-level filtering, content pattern filtering); (4) logarithmic transformation of like counts — to address the right-skewed long-tail distribution, likes were transformed using log(likes + 1) to compress the influence of extreme values and improve approximate normality; (5) special treatment of timestamp data — since Xiaohongshu's data provides only publication year without exact dates, this portion of the sample was explicitly excluded from the time-series analysis to ensure the precision of temporal analyses.

For the validity assessment of RoBERTa sentiment analysis, we have added a two-level reliability check procedure to Section 3.2 of the revised manuscript. The first level is overall random verification: 300 texts were randomly sampled from the 3,691 texts, and two authors independently judged their sentiment polarity and compared the results with RoBERTa's output to establish an overall agreement rate as a baseline indicator of model performance. The second level is targeted irony verification: given the particular challenges that ironic and metaphorical expressions — prevalent in Chinese online discourse — pose to sentiment analysis, we specifically sampled 300 texts from those classified as "positive" by RoBERTa for targeted verification, with results showing that the model achieved a positive-sentiment classification accuracy of 94.67%. This two-level check assesses model performance not only on general samples but also specifically on irony-prone samples. For the small remaining proportion of irony misclassifications, we make an explicit statement in the limitations section, while emphasizing that the core findings of this study rely primarily on relative sentiment differences across topics, geographic regions, and temporal dimensions — structural findings that are sufficiently robust to a small number of irony misclassifications.

Regarding analytical coherence, we have ensured the internal consistency of the inferential chain from data to conclusions through three mechanisms in the revised manuscript: (1) one-to-one correspondence between research questions and analytical methods; (2) one-to-one correspondence between the theoretical framework and empirical findings, as articulated in the newly added Section 2.4; and (3) explicit separation of levels of interpretation — in the discussion section, we systematically distinguish between "empirical findings" (descriptive facts directly supported by the data, expressed in affirmative language) and "theoretical interpretations" (interpretations of the data based on theoretical frameworks, expressed in cautious language such as "may," "may be understood as," and "warrants further verification"), avoiding interpretive overreach.

*6) Describe the pipeline. Present the structured results (discursive, emotional, and resonance levels) with examples of quotes. Specify which software and methods were used for the qualitative analysis of discourse and sentiment. Specify the version of the ROBERTa model; the techniques for feedback analysis (resonance); the metrics and other statistical methods used.*

We sincerely thank the reviewer for these systematic suggestions regarding the presentation of methods and results. We fully agree that a study relying on computational methods should provide sufficient operational information at four levels — the analysis pipeline, the structured presentation of results, software and model versions, and specific technical details — to ensure that readers can clearly understand and, if necessary, reproduce the complete analytical process. We wish to respectfully note that the various elements listed in this comment are closely aligned with the concerns raised by other reviewers regarding "insufficient methodological detail," "automated classification reliability," and "ensuring reproducibility." To systematically address these interconnected concerns, we have completely rewritten the methods section (Section 3), specifying details down to individual parameter settings; and we have rewritten the topic extraction table in Section 4, adding representative keyword examples for each theme.

*7) The data and materials available at https://osf.io/eu6mp/files/xeagu are incomplete; the initial data, data from the NLP processing pipeline, and model output data must be presented.*

We have reorganized the data repository content and URL. The raw data, code, and process-level model output data are now presented within a larger folder. The new repository URL is: https://doi.org/10.17605/OSF.IO/CA42G

**Reviewer #2:**

*First, the theoretical integration between occupational burnout, emotional labor, and affective publics could be articulated more clearly. While the literature review introduces these frameworks individually, the manuscript would benefit from a more explicit explanation of how they jointly inform the research questions and analytical strategy. Clarifying the conceptual pathway that connects individual emotional experiences, digital discourse, and collective resonance would help strengthen the coherence of the theoretical contribution.*

We sincerely thank the reviewer for this constructive suggestion. We fully agree that, although the original manuscript's literature review introduced the three theoretical frameworks of occupational burnout, emotional labor, and affective publics individually, it did not explicitly articulate how they jointly serve the study's core questions, nor did it clearly present the conceptual chain running from individual emotional experience through digital discursive practice to collective resonance mechanisms. This expressive gap diminished the logical coherence of the paper's theoretical contribution, and we sincerely apologize for this. We have made corresponding additions in the revised manuscript.

We have provided a clearer differentiation of the theoretical roles each of the three frameworks plays in this study. (A) Occupational burnout theory (in particular the JD-R model and Maslach's three-dimensional framework) provides the study's definition of its research object — it tells us "what burnout is" and "what dimensions burnout has," constituting the basic reference system for understanding burnout expressions in social media discourse. However, this traditional framework presupposes burnout as an individual psychological state measurable by scales, whereas this study deals with burnout discourse spontaneously produced by employees in digital public spaces, necessitating the other two theoretical frameworks to supplement its explanatory power. (B) Emotional labor theory (Hochschild and subsequent scholars) provides explanatory tools at the level of individual experience, enabling us to understand employees' emotion regulation practices under the constraints of organizational display rules, as well as the resistant affective practices these rules provoke when they become unreasonable. This framework primarily serves the theoretical interpretation of RQ1 (discourse) and RQ2 (sentiment). (C) Affective publics theory (Papacharissi and related scholarship) provides explanatory tools at the collective level, enabling us to understand why certain affective expressions generate resonance and circulation in digital public spaces, and how such circulation shapes temporary yet powerful collective identities. This framework primarily serves the theoretical interpretation of RQ3 (resonance). In other words, the three frameworks in this study do not operate in parallel but constitute an analytical chain moving from micro to macro: burnout theory defines the research object → emotional labor theory explains how individuals experience and express burnout in organizational contexts → affective publics theory explains how these individual expressions coalesce into collective affective practices in digital spaces.

Based on this approach, we have added a subsection — "2.4 Theoretical Integration and Analytical Approach" (approximately one page) — at the end of Section 2 of the literature review, explicitly articulating the interrelationship and division of labor among the three theoretical frameworks, and mapping this integrated framework onto the three research questions (RQ1–RQ3). The reviewer's comment has in fact prompted us to make an improvement that goes beyond "expressive clarification" — it causes the theoretical logic that was previously implicit in the discussion section to be explicitly presented to readers at the literature review stage, thereby upgrading the paper's theoretical architecture from "three parallel frameworks" to "one organically integrated analytical system."

*Second, the methodological section would benefit from additional detail regarding the data collection and preprocessing procedures. For example, providing clearer information about the keyword selection process, inclusion and exclusion criteria for posts, handling of duplicate or automated content, and the overall sampling logic would improve reproducibility. Given that the study relies heavily on automated text classification and sentiment analysis, a more detailed explanation of the validation procedures used to assess the reliability of the classification results would also be valuable.*

We sincerely thank the reviewer for this concern regarding methodological transparency. We fully agree that in research relying on automated text classification and sentiment analysis, thorough presentation of data collection, preprocessing, and reliability validation procedures is crucial for reproducibility and credibility. The original manuscript's presentation of these details was insufficiently systematic, with many operational steps scattered across different parts of the methods section without forming a clear procedural account. We sincerely apologize for this and have made systematic additions to Section 3 of the revised manuscript.

The revisions encompass the following five aspects. (1) Keyword selection: the revised manuscript adds a paragraph in Section 3.1 explicitly stating that keyword selection adopted a two-stage "theory-driven plus data-driven" strategy. (2) Inclusion and exclusion criteria for posts: a clear inclusion/exclusion criteria statement has been added in Section 3.1. (3) Handling of duplicate and automated content: a paragraph detailing the specific steps of deduplication and anti-bot processing has been added to Section 3.1. (4) Overall sampling logic: a paragraph explicitly stating the overall sampling strategy has been added to Section 3.1. (5) Reliability validation procedures for automated classification and sentiment analysis: more detailed reliability validation procedures have been supplemented in Sections 3.2 and 3.3 respectively — for BERTopic + LLM topic classification, the paragraph on reliability validation in Section 3.2 has been expanded to explicitly describe the three-step validation procedure; for RoBERTa sentiment analysis, a paragraph on sentiment classification reliability has been added to Section 3.3.

*Third, some interpretations presented in the discussion appear to extend beyond what can be directly supported by the empirical findings. While the interpretations regarding themes such as "institutional absurdity" and "internal friction resistance" are insightful, presenting these interpretations with slightly greater caution and clearly distinguishing between empirical evidence and theoretical inference would strengthen the analytical balance of the manuscript. This is particularly relevant in the discussion of geographic variation, where the proposed explanations relating to economic transitions and cultural patterns may be plausible but would likely require additional empirical evidence to be fully substantiated.*

We sincerely thank the reviewer for this precise and pertinent comment. We fully agree that in the discussion section, the original manuscript's interpretations of certain themes (particularly "Institutional Absurdity" and "Internal Friction Resistance") and of geographic variation extended beyond what the empirical data can directly support. This tendency for "interpretive ambition to exceed data support" diminished the analytical balance of the paper, and we sincerely apologize for this. We have made systematic tonal adjustments and boundary redrawn in the revised manuscript.

Regarding the revision of thematic interpretations of "Institutional Absurdity" and "Internal Friction Resistance": we fully accept the reviewer's judgment that the original manuscript's interpretations of these themes in Sections 5.1 and 5.3 (for example, "Institutional Absurdity generates resonance because of users' shared recognition and ironic deconstruction of the system's irrational logic") in fact contain a researcher-led interpretive leap from "high-engagement data" to "collective cognitive motivation." The textual and engagement data can only tell us that this type of content received higher engagement; they cannot directly reveal why users liked, shared, or commented. To this end, we have substantially rewritten this passage in Section 5.3 of the revised manuscript, explicitly repositioning it as "one possible theoretical interpretation" and proactively listing several alternative explanations equally compatible with the data, including: the entertainment hypothesis, the algorithmic amplification hypothesis, and the safe catharsis hypothesis. These four explanatory mechanisms are not mutually exclusive, and none can be fully ruled out based on the cross-sectional textual data. We explicitly acknowledge this in the revised manuscript and have systematically replaced the original affirmative language with more cautious expressions. We simultaneously emphasize that even if no adjudication can be made among these explanations, the empirical finding that "content categorized under 'Institutional Absurdity' demonstrates significantly greater circulatory power in Chinese digital workplace discourse than other thematic categories" remains valid and continues to constitute a meaningful empirical contribution to affective publics theory in the context of Chinese workplace issues.

The reviewer's comment is particularly applicable to the discussion of "emotional lowland" and "emotional relief zone" in Section 5.2, and we fully accept this judgment. The explanations offered in the original manuscript for regional variation — drawing on the JD-R model and cultural psychology theories of collectivism/individualism — in fact failed to control for key confounding factors such as platform usage habits, age structure, industry distribution, online linguistic style, and region-specific biases in the sentiment analysis model's recognition capacity, and the IP-registered location data used in this study do not provide the conditions necessary to control for these factors. To this end, we have added a declaration at the beginning of Section 5.2 of the revised manuscript, explicitly repositioning "emotional lowland" and "emotional relief zone" as descriptive labels for the observed data distribution rather than substantive characterizations of the authentic emotional states of workers in these regions; we also explicitly state that the subsequent discussion should be understood as a phenomenological description of spatial distribution and a set of hypothetical interpretations rather than conclusive claims about inter-regional differences in burnout. We have also systematically replaced causal or affirmative language in the original manuscript with cautious expressions such as "may be accompanied by," "one possible but as yet empirically unverified interpretation is," and "may tend toward."

*Finally, the manuscript could benefit from a more developed reflection on the limitations associated with social media data. Although the authors briefly acknowledge that the dataset primarily represents younger and digitally active populations, further discussion of potential biases related to platform dynamics, self-selection of users, and algorithmic amplification would provide useful context for interpreting the findings. Expanding this section would help situate the results within the broader methodological challenges of digital trace data.*

We sincerely thank the reviewer for this constructive suggestion regarding the discussion of data source limitations. We fully agree that the original manuscript's reflection on social media data in the limitations section was overly brief. We wish to note that the three aspects raised in the reviewer's comment are also addressed in our responses to other reviewers' comments: the discussion of user self-selection and representativeness has been addressed in detail in the response to the comment on "data representativeness"; the impact of algorithmic amplification on the interpretation of resonance has been explicitly incorporated into the revision of Section 5.3 (as one of the alternative explanations) in the response to the comment on "causality and overinterpretation"; and platform censorship and self-censorship have been systematically addressed in the epistemological declaration on the data production process added at the end of Section 3.1 of the revised manuscript. The expanded reflection on social media data limitations in this revision is therefore a systematic cross-chapter revision rather than a single-point addition confined to the conclusions section.

**Reviewer #3:**

*1. The title does not accurately reflect the data analysis and the results obtained.*

We fully accept the reviewer's judgment. The original title "What Burns You Out? Discourse, Sentiment and Resonance in Chinese Workplace" used a causal interrogation ("what makes you burned out") as its main title, in effect promising the identification and explanation of the causes of burnout. However, as the reviewer astutely points out, the actual analytical path of this study is the thematic mining, sentiment modeling, and resonance pattern identification of burnout discourse in Chinese social media corpora. We answer "how employees talk about burnout," "what affective and spatiotemporal characteristics these expressions exhibit," and "which expressions most readily generate collective resonance" — not "what factors cause burnout." These two types of questions differ importantly at the epistemological level, and the original title's phrasing could indeed generate misleading expectations for readers.

After careful consideration, we have revised the title to: Discourse, Sentiment, and Resonance of Workplace Burnout on Chinese Social Media: A Computational Analysis. This new title more accurately reflects the paper's actual content in three respects: (1) "Discourse, Sentiment, and Resonance" directly corresponds to the three research questions (RQ1–RQ3) and the tripartite analytical structure, making the research framework immediately transparent; (2) "Workplace Burnout on Chinese Social Media" clearly delineates the boundaries of the research object — we examine burnout discourse practices on social media rather than burnout itself as a clinical or organizational-psychological measurement, avoiding the overgeneralization that the original title might invite; (3) "A Computational Analysis" faithfully represents our methodological approach, namely computational methods centered on BERTopic topic modeling, RoBERTa sentiment analysis, and LLM-assisted coding.

*2. What was the purpose of including a subsection on emotional labor in the section on the literature review and theoretical framework, given that this issue was omitted from the analysis and discussion?*

We sincerely thank the reviewer for this precise observation. This comment prompted us to reexamine the argumentative trajectory of the paper, and we sincerely accept this criticism. We acknowledge that the theoretical connection between Section 2.2 of the literature review and the subsequent analysis was not clearly signposted in the original manuscript, giving the reviewer the impression that "the emotional labor issue was set aside in the analysis." We sincerely apologize for this.

However, we wish to respectfully invite the reviewer to note Section 5.1, "The Shift in Emotional Labor: A Battle for Emotional Sovereignty." In fact, emotional labor theory is the core analytical lens through which that section interprets the empirical findings: (a) we reinterpret "Internal Friction Resistance" and "Clear Detachment" as a new form of emotional labor — "a performance of non-performance" — thereby extending Hochschild's classic surface/deep acting dichotomy; (b) we draw on themes such as "Workplace Humiliation," "Management Disillusionment," and "Institutional Absurdity" to argue that contemporary display rules have shifted from "requiring the display of positive emotions" to "being characterized by negative emotional disciplining," thereby elevating emotional labor from an individual coping strategy to a matter of organizational justice; (c) through "Time Deprivation" and "Task Overload," we argue that the failure of emotional labor is often not due to individual incapacity but to the structural deprivation of the time and cognitive resources upon which deep acting depends; (d) through "Midlife Cliff" and "Professional Burnout," we reveal the cumulative and life-course dimensions of the costs of emotional labor. These four points are consolidated in Figure 5, which aims to advance emotional labor theory from the framework of "individual adaptation to organizational rules" to a new paradigm of "subjects reconstructing emotional autonomy under institutional pressure."

If a specialist reader such as the reviewer was unable to clearly perceive this theoretical continuity, the responsibility lies with our writing rather than with the reviewer's reading. To this end, we have made the following revisions to the manuscript to enhance visibility: (1) a transitional sentence has been added at the end of Section 2.2 explicitly announcing that emotional labor theory will be revisited and extended in the discussion section in conjunction with the empirical findings; (2) Section 5.1 now explicitly back-references the key concepts introduced in Section 2.2 (surface acting, deep acting, display rules, emotional dissonance), making the theoretical dialogue clearly legible; (3) the opening paragraph of Section 5.1 has been rewritten to directly declare that this section represents the paper's core theoretical contribution to the emotional labor literature.

*3. Data and Sampling Limitations: The sample of 3,691 social media posts may not reflect the broader population of workers in China (overrepresentation of younger, urban, digitally active users). Results may depend on the specific platform (e.g., censorship, algorithms promoting certain types of content). Individuals experiencing stronger burnout are more likely to post, potentially inflating negative sentiment.*

We sincerely thank the reviewer for identifying these three important limitations at the data and sampling level. These issues are indeed the methodological challenges that computational social science research based on social media corpora commonly faces, and we fully accept the reviewer's judgment.

Regarding the sampling bias toward younger, urban, and digitally active populations: we fully acknowledge that this bias objectively exists and cannot be eliminated through post-hoc weighting or sampling adjustment. However, we wish to respectfully note that this bias does not necessarily diminish the study's theoretical contribution for two reasons. First, the study's research questions are not "what is the level of burnout among all Chinese workers," but rather "how, in digital public spaces, is burnout discursivized, affectivized, and resonated." The latter is fundamentally a question about digital affective publicity (resonating with Papacharissi's affective publics theory), whose legitimate field of study is precisely these populations active on social media — they are the actual participants and shapers of digital affective mobilization. Second, while urban young internet users cannot represent all workers, they are precisely the most active producers and disseminators of current Chinese workplace burnout discourse, and the originating population of key cultural symbols such as "involution," "lying flat," and "996." Positioning the sample boundary as "digitally active urban young workers" rather than "Chinese workers" is thus a more honest and accurate delineation of the research scope. Fortunately, we have already revised the title to limit the description of the research scope to "social media," which should prevent readers from misunderstanding.

Regarding the potential impact of platform-specific characteristics: this is indeed a profound issue that is difficult to fully resolve. We acknowledge that platform censorship may suppress certain extreme expressions, while recommendation algorithms may amplify the visibility of certain types of content — meaning that the "high-resonance" content we observe is to some extent a co-product of platform logic. To address this, we have taken two mitigation measures. First, cross-platform sampling: our corpus comes from five platforms — Xiaohongshu, Bilibili, Weibo, Douyin, and Douban — with different user structures, censorship intensities, and recommendation mechanisms. This cross-platform design disperses to some extent the biases of any single platform's logic. Second, we have added a passage in Section 5.3 of the revised manuscript explicitly noting that the finding of "high resonance for 'Institutional Absurdity' content" must be understood in the context of "algorithmic mediation": what we observe is not pure user preference but the product of the interaction between user preferences and platform recommendation mechanisms — which is precisely what Papacharissi's (2016) theory of affective publics means by "algorithmically mediated publicness," rather than a source of data contamination to be purged.

Regarding the self-selection bias by which "higher-burnout individuals are more likely to post," potentially inflating negative sentiment: this bias does exist, and our overall negative sentiment mean (−0.191) may to some extent reflect this bias rather than the authentic emotional baseline of Chinese workers. We wish to note that the core claims of this study do not depend on the metric of "absolute sentiment level." We do not claim that "Chinese workers as a whole are in a negative emotional state." Our key findings focus on relative differences and structural patterns: differential resonance across themes (RQ3), sentiment gradients across regions (RQ2b), and cyclical fluctuations over time (RQ2a). These relative findings are sufficiently robust to mean shifts. We have explicitly acknowledged in the limitations section that absolute sentiment values should not be interpreted as estimates of the overall emotional state of Chinese workers, but only as a description of the sentiment distribution among those who actively post on social media.

*4. Issues with Concept Operationalization: ("internal friction resistance" or "institutional absurdity"). Risk of overinterpreting colloquial language as coherent theoretical categories.*

We sincerely thank the reviewer for raising this important methodological critique. "Overinterpreting colloquial expressions as coherent theoretical categories" is indeed a core risk that research employing unsupervised topic modeling methods such as BERTopic commonly faces. We first frankly acknowledge that from "high-frequency co-occurrence word clusters" to naming such as "Internal Friction Resistance" or "Institutional Absurdity," there is inevitably a researcher-led conceptualization leap. If this leap lacks procedural constraints, it may degenerate into researcher subjective projection. The reviewer's criticism therefore strikes at the methodological vulnerability of BERTopic-type methods. In the original manuscript, we in fact designed three safeguard mechanisms to constrain this interpretive leap, but their details were not sufficiently highlighted in the original text.

First, theme naming is directly supported by the semantic core of representative keywords rather than by researcher post-hoc interpretation. We respectfully invite the reviewer to consult the representative keyword clusters for each theme in Table 1 of the revised manuscript — they are not fragmented TF-IDF noise but semantically highly convergent discourse clusters. We believe that the representative keywords presented in the new Table 1 are sufficient to demonstrate to readers that theme naming is an induction of the already-existing semantic core of word clusters rather than arbitrary imposition.

Second, LLM coding serves as an independent secondary validation. After determining themes and their names, we used the Qwen-Plus model to reclassify the entire sample. The crucial point is that the LLM classification was based not on representative keywords but on the topic definitions we provided. If our theme naming were merely a forced association of fragmented keywords by researchers, the LLM would be unable to reliably assign a new text to the correct category. In fact, the agreement between LLM classification results and human verification reached Fleiss's Kappa = 0.7827, indicating that the topic definitions are semantically stable entities that can be reliably identified by independent judges, not researcher subjective projections.

Third, theme naming follows the principle of in vivo coding, mapping onto indigenous categories already circulating in Chinese online spaces. We acknowledge that some theme names (such as "Internal Friction Resistance" and "Clear Detachment") borrow existing discourse labels, but this is not arbitrary appropriation. These labels are indigenous concepts spontaneously invented and widely used by Chinese workers in digital public spaces — for example, the term "internal friction (neihao)" appears millions of times in Douban groups and Xiaohongshu topics and has become a consensual indigenous affective term. Our work merely maps these indigenous categories already consolidated among user communities onto academic theories such as emotional labor and affective publics, rather than constructing categories ex nihilo and imposing them on the data. This approach is referred to in qualitative research methodology as in vivo coding, whose methodological legitimacy lies precisely in respecting the conceptual systems of the research subjects themselves.

Even so, we fully accept that the risk highlighted by the reviewer warrants greater caution in expression. To this end, we have made the following adjustments in the revised manuscript: (1) Section 3.2 explicitly distinguishes between "BERTopic's statistical clustering output" and "the researcher's conceptualization and naming based on full texts" as two separate steps; (2) the "Representative Keywords" column in Table 1 has been revised to present more semantically representative complete keyword clusters rather than partially fragmented keywords from the original manuscript; (3) a sentence has been added in the limitations section explicitly positioning the study's findings as "descriptions of, and theoretical mappings onto, indigenous affective categories that can be stably identified within Chinese digital workplace discourse" rather than "discoveries of objectively pre-existing categories."

*5. Limitations of NLP Methods: (BERTopic — depend on model parameters and may be unstable). RoBERTa (sentiment analysis — struggle with: irony, sarcasm etc.), LLM-assisted coding: limited transparency in the coding process)*

We fully agree that BERTopic's output is sensitive to the hyperparameters of UMAP and HDBSCAN (in particular n_neighbors, min_cluster_size, min_dist, and others), and that different parameter combinations may produce different clustering structures. This is a common issue with unsupervised topic modeling and is the inherent cost of BERTopic as a "data-driven" method. In the original manuscript, we took three measures to mitigate this risk: first, fixing the random seed to ensure reproducibility of the UMAP dimensionality reduction stage; second, using LLM coding as an independent secondary validation — even if BERTopic's initial clustering exhibits parameter dependence, we did not use it directly as the final classification result; rather, we derived topic definitions from the clustering results and then used Qwen-Plus to reclassify all texts based on those definitions, so that the final topic assignments no longer depend entirely on a single BERTopic run; third, human verification and Kappa inter-rater agreement (Fleiss's Kappa = 0.7827) provide external reliability guarantees for the final classification results. Although a single BERTopic run is parameter-sensitive, our overall research pipeline constrains this sensitivity to the first stage (exploratory clustering) through a two-stage design, with the final conclusions depending on the externally verifiable second stage (definition-based LLM + human coding). We have supplemented Section 3.2 of the revised manuscript with detailed parameter configuration information and have provided process-level data.

Regarding the limitations of RoBERTa sentiment analysis in recognizing irony and metaphor: this is a problem we proactively acknowledged in the limitations section of the original manuscript's conclusions, and the reviewer's reminder further reinforces the necessity of taking this limitation seriously. Ironic expressions in Chinese online contexts — for example, "only working one month a year," "thank the company for sparing my life," "grateful to my boss for the life experience" — may appear positive at the literal level while actually expressing strongly negative sentiment. Any sentiment classifier based on a pretrained language model will struggle to fully capture this pragmatic reversal in an unsupervised setting, and RoBERTa is no exception. Irony recognition failure primarily affects the precision of absolute sentiment mean values rather than the structural findings of relative differences. The core claims of this study focus on relative patterns: differential resonance across themes (RQ3), sentiment gradients across regions (RQ2b), and cyclical fluctuations over time (RQ2a). We have quantitatively estimated the irony misclassification rate as a robustness check: 300 texts classified as "positive" by RoBERTa were randomly sampled for human verification (accuracy = 94.67%), identifying the proportion of ironic expressions misclassified by the model. This supplementary analysis allows reviewers and readers to directly assess the actual impact of irony recognition failure on the conclusions.

Regarding the limited transparency in the LLM-assisted coding process: we fully accept this concern and have implemented three safeguards to enhance process transparency. First, complete disclosure of prompt engineering: the system prompt used to guide Qwen-Plus in classification is fully standardized and structured, containing explicit definitions and behavioral boundaries for each thematic category. The complete content of this prompt has been uploaded as supplementary material to the OSF open-access repository, allowing any researcher to reproduce our coding procedure using exactly the same prompt. Second, the temperature parameter was set to 0 to ensure output determinism: this setting eliminates the randomness of LLM output, ensuring that identical inputs always produce identical outputs, thereby constraining the "black box" to a deterministic function. Third, human verification of 10% of the sample serves as external calibration, with agreement between LLM coding results and human coding reaching Fleiss's Kappa = 0.7827. This process essentially externally aligns the LLM's "black-box reasoning" with the "traceable reasoning" of human coding. Although we cannot inspect the LLM's internal process, we can demonstrate that its output converges highly with the judgments of trained human coders, providing result-level reliability even in the absence of process-level interpretability.

*6. Lack of Methodological Triangulation (Reliance solely on social media text: no interviews, qualitative validation, or organizational data.)*

We sincerely thank the reviewer for raising this important concern regarding methodological triangulation. We have carefully considered this criticism and wish to respond frankly. We fully agree that, from the general principles of research methodology, combining interviews, qualitative validation, or organizational internal data could indeed provide valuable triangulation for social media text analysis. Research relying on a single data source does need to exercise greater caution when generalizing conclusions. The reviewer's criticism is therefore pertinent and reasonable. However, after careful deliberation, we have decided not to supplement this manuscript with qualitative interviews or organizational data, but instead to address this limitation by more rigorously delineating the epistemological boundaries of the study.

This decision is based on the following considerations. First, supplementing qualitative methods would substantially alter the positioning and structure of this study. Symbolically adding a small number of interviews (e.g., 8–10 participants) merely to respond to reviewer comments would not support rigorous triangulation and might instead undermine the study's methodological integrity by appearing perfunctory. A mixed-methods study of adequate scale (e.g., systematic interviews with 20–30 participants plus coding) would require months of additional fieldwork and a complete IRB review process, exceeding what can reasonably be undertaken within a single revision, and would substantially transform this paper from a "computational social science study" into a "mixed-methods study" — two types with genuine differences in research design, theoretical contribution, and methodological commitment. Second, the more honest approach is to clearly delineate the study's boundaries as a purely computational analysis.

Based on this approach, we have made the following key adjustments in the revised manuscript. (1) The title has been revised to clearly specify the study's methodological nature and data boundaries, with "A Computational Analysis" in the subtitle explicitly informing readers of the methodological type and the main title strictly confining the research object to "workplace burnout on Chinese social media" rather than burnout experience per se. (2) In the limitations section, we have added a passage frankly acknowledging the limitations of a single data source and explicitly designating "mixed-methods research combining in-depth interviews, organizational internal data, and longitudinal tracking" as an important direction for future research. (3) For explanatory inferences relying on external theory, we have further strengthened the "inferential rather than affirmative" tone, repeatedly employing language such as "may," "may perhaps reflect," and "awaits further empirical verification."

We respectfully wish to note that computational analysis based on social media texts itself constitutes a research approach with independent methodological legitimacy, rather than a secondary method that requires supplementation by interviews or organizational data to be valid. Papacharissi's (2016) foundational research on affective publics and the large body of recent computational social science research based on Twitter/Weibo are built on single data sources, deriving their legitimacy from the intrinsic alignment between the research questions and the data source — the researchers inquire about discursive practices in digital public spaces, and those practices naturally occur on social media itself. The three core questions of this study (how employees talk about burnout, what affective and spatiotemporal patterns these expressions exhibit, and which expressions most readily generate collective resonance) belong to precisely this category. We believe that honestly acknowledging the boundaries of the data source and correspondingly delineating the scope of the conclusions — rather than reluctantly supplementing an insufficiently scaled qualitative study — more genuinely responds to the reviewer's concern, because it allows readers from the outset to know clearly what they will and will not find in the paper.

*7. Causality Issues: The study is descriptive and exploratory, cannot establish what actually causes burnout. Lack of deeper causal analysis in the dynamics of emotions and resonance. The conclusion describes these phenomena but does not sufficiently explore their causes in the context of the collected data. For example, why does "institutional absurdity" resonate the most? The study points to "shared recognition and ironic deconstruction of systemic irrational logic," but do the textual data actually allow for such a clear interpretation of motivation? Is this the only or primary cause?*

Regarding the overall causal positioning of the study: we fully accept the reviewer's judgment that this study is descriptive and exploratory in its methodology and does not possess the capacity to establish the causal proposition "what causes burnout." Based on cross-sectional social media text data, no matter how refined the analytical methods, the causal mechanisms of burnout formation cannot be revealed, as this requires longitudinal tracking, organizational-level comparative designs, or quasi-experimental methods. We have made the following explicit delineations in the revised manuscript: (1) the title has been revised to "A Computational Analysis," clearly defining the methodological nature of the study as a computational description and interpretation of social media discourse rather than causal inference; (2) the wording of the research questions (RQ1–RQ3) in the original manuscript uses "how," "what," and "which" rather than "why" or "what causes" — we deliberately avoided any causal terminology; (3) a passage has been added to the limitations section in the conclusion, frankly acknowledging that this study cannot establish causal mechanisms of burnout and explicitly designating "revealing the causal chain of burnout through longitudinal tracking, organizational-level comparative designs, or quasi-experimental methods" as a direction for future research.

Regarding the specific challenge to the explanation of "Institutional Absurdity"'s high resonance: this is the sharpest and most worthy of serious reflection point in the reviewer's comments. The reviewer correctly notes that textual data alone can only tell us that this type of content received higher engagement; it cannot directly reveal why users liked, shared, or commented. From "high engagement" to "collective recognition and ironic deconstruction" there is indeed a researcher-led interpretive leap. We apologize for this overreach. After reflection, we recognize that the high resonance of "Institutional Absurdity" admits at least the following possible explanatory mechanisms, all compatible with the existing data. (A) The cognitive consensus hypothesis (the explanation the original manuscript advances): Institutional Absurdity content, by exposing the rupture between organizational rules and everyday experience, activates users' consensual cognition, generating collective identification of "I feel the same way." (B) The entertainment hypothesis: Institutional Absurdity content makes extensive use of mockery, self-deprecation, and ironic rhetoric, and its engagement advantage may partly derive from its entertainment value rather than its critical depth — users' likes may reflect amusement rather than a sense of recognition. (C) The algorithmic amplification hypothesis: Institutional Absurdity content tends to have textual features more conducive to circulation (concise, conflict-laden, easily screenshot and shared), and platform recommendation algorithms may systematically amplify the visibility of such content, producing an endogenous inflation of engagement metrics rather than a true reflection of user preferences. (D) The safe catharsis hypothesis: compared to content that directly criticizes specific companies or individuals (such as "Workplace Humiliation" content), "Institutional Absurdity" expresses dissatisfaction in a systemic rather than targeted manner, lowering the cost of engagement for users, since liking or sharing carries no direct interpersonal or professional risk. These four explanations are not mutually exclusive, and none can be fully ruled out based on the available textual and engagement data. The original manuscript presented only the first explanation (the cognitive consensus hypothesis) and expressed it as the sole or primary cause, which indeed exceeded the inferential scope supportable by the data. We have substantially rewritten that section, repositioning the original affirmative claim as "one possible theoretical interpretation" and explicitly listing the alternative explanations above, acknowledging that the existing data cannot adjudicate among them. This rewriting is intended to retain the original manuscript's theoretical sensitivity and interpretive insight while repositioning it as a possible reading grounded in affective publics theory rather than a causal claim about underlying mechanisms.

*8. Spatial Interpretation Concerns: "emotional lowland" and "relief zone" oversimplification, does not control for confounding factors (e.g., platform usage, age, language differences, industry distribution).*

We sincerely thank the reviewer for this specific and pertinent critique of the spatial analysis section. The reviewer identifies two interrelated problems: first, the descriptive labels "emotional lowland" and "emotional relief zone" risk oversimplification; second, the explanations offered for regional affective variation (economic transition pressures and cultural-psychological mechanisms) fail to control for key confounding factors such as platform usage habits, age, language differences, and industry distribution. We must acknowledge that we cannot control for any of these confounding factors through the available data — IP-registered location is the only geographic variable we can obtain, and the sample does not contain user age, industry, or more granular demographic information. This fundamental data-level limitation means that any explanation of regional variation must maintain a high degree of caution.

We wish to respectfully note, however, that the original manuscript in fact already made an explicit statement at the beginning of Section 5.2: "Based on the observation of the spatial distribution of occupational burnout levels, this study draws preliminary inferences: such regional differences may result not only from varying levels of economic development but also from the combined effects of socioeconomic structural imbalances and cultural-psychological mechanisms. The following discussion unfolds along two dimensions, with the caveat that these inferences offer only a possible theoretical explanation and require further empirical validation." We had originally intended to position this section as hypothesis generation rather than data-based conclusive claims.

Nevertheless, we fully accept the reviewer's judgment. The cautious phrasing of the original manuscript is insufficient to counteract the reifying suggestion carried by geographic labels such as "emotional lowland" and "emotional relief zone" themselves. When readers see a map colored red, yellow, and green and read terms such as "emotional lowland," the visual and naming impact may overwhelm the qualifying language even if the text repeatedly emphasizes "inference." We apologize for this risk.

Based on this reflection, we have made the following substantive revisions in the revised manuscript. First, "emotional lowland" and "emotional relief zone" have been repositioned as purely descriptive labels, with explicit qualifying declarations added upon first appearance (in Section 4.2) in quotation marks. Second, the causal language in Sections 5.2(a) and 5.2(b) has been substantially softened, with systematic replacement of expressions that remain overly affirmative in the original manuscript. Third, an explicit statement of the limitations of the spatial analysis has been added to the limitations section, to prevent readers from deriving the oversimplified impression from the abstract or conclusion alone that "this study found inter-regional differences in burnout."

*9. Political and Cultural Context. In China censorship and self-censorship may distort the data, users may rely on metaphor or irony, that models misinterpret. Lack of cultural context in interpreting "emotional exhaustion": There is insufficient context regarding what "traditional individualized narratives" mean within Chinese culture. Did "emotional exhaustion" carry specific cultural connotations that are now changing? Without this context, the claim of a "shift from passive endurance to active boundary-setting" may be difficult to fully interpret for readers unfamiliar with the nuances of the Chinese work environment.*

We sincerely thank the reviewer for raising these two concerns that are of critical importance for cross-cultural academic communication.

Regarding censorship and self-censorship: we fully agree that platform content moderation and user self-censorship in the Chinese social media context may produce systematic effects on the textual data we collected, potentially operating at two levels. At the direct censorship level, sharp criticism targeting specific companies, specific managers, or specific events is more likely to be deleted or de-ranked by platforms, meaning our corpus may systematically lack the most intense, explicitly targeted protest expressions. The workplace burnout discourse we observe may already be a platform-filtered, moderated version, as the most genuinely angry and action-oriented expressions may have been deleted before we could crawl them. At the self-censorship level, users anticipating censorship actively choose expressive strategies such as metaphor, irony, wordplay, and phonetic substitutions to circumvent risk. This is precisely an important linguistic phenomenon observed in this study: the prevalence of internet terms such as "oxen and horses (niuma)," "corporate livestock (shezhu)," "workers (dagong ren)," "doge," "emmm," and "ctbz" is itself a product of discursive strategies under conditions of self-censorship. Interestingly, this phenomenon is simultaneously a limitation of this study (irony and metaphor are difficult for models to accurately recognize) and a finding of the study itself (the discursive strategy of using satirical rhetoric to deconstruct serious issues in the "Institutional Absurdity" theme reflects the distinctive form of collective affective mobilization in constrained expressive spaces). We have added a clear declaration in the methods section (Section 3) of the revised manuscript, acknowledging censorship and self-censorship as part of the data production process, and have explicitly listed "the burnout discourse observed in this study is already the product of platform-filtered expression, which may underestimate the intensity and specificity of authentic sentiment" as an explicit validity boundary in the limitations section.

Regarding insufficient cultural context for international readers: we sincerely thank the reviewer for reminding us that the series of theoretical claims made in this study presuppose that readers are already familiar with the background knowledge of contemporary Chinese workplace culture, which international readers may not be, and may therefore be unable to fully evaluate the weight and plausibility of our claims. We sincerely apologize for this and fully accept this criticism. In the revised manuscript, we have added a diachronic cultural background passage in the introduction, after the second paragraph and before the statement of the research questions — a position where the text was already discussing "the constraints on formal communication channels in the Chinese context," making a paragraph of diachronic cultural background an apt transition. This passage explains to readers the transformation in Chinese workplace discourse since around 2010 from "individual attribution" to "structural attribution," and in particular explains the semantic evolution of indigenous terms such as "involution," "lying flat," and "internal friction" along with their scholarly interpretations, including anthropologist Xiang Biao's classic definition of "involution" as "an endless competitive cycle in which individuals know it to be meaningless yet cannot exit." This addition grounds the study's central claim — that "Chinese workplace discourse is undergoing a paradigm shift from passive endurance to active boundary-setting" — in an empirically observable diachronic trajectory, enabling international readers to form an accurate contextual understanding of the specific claim before encountering it in the findings and discussion.
