# Supplementary material for: Discourse, sentiment, and resonance of workplace burnout on Chinese social media: A computational analysis
Source: PLoS One. 2026 Aug 5;21(8):e0346396. doi: 10.1371/journal.pone.0346396 (PMC13440837; doi:10.1371/journal.pone.0346396)
Supplement: Supplementary file 2 [file pone.0346396.s006.docx]

**Response to Reviewers**

Manuscript: PONE-D-26-05195R2

*Discourse, Sentiment, and Resonance of Workplace Burnout on Chinese Social Media: A Computational Analysis*

We sincerely thank the Academic Editor and both reviewers for their careful and constructive engagement with this manuscript. Reviewer #1 now recommends acceptance and Reviewer #4 has already recommended acceptance, and we are grateful for the editorial guidance that remains. Below we provide a point-by-point response to all comments raised in this round. All changes are highlighted in the Revised Manuscript with Track Changes.

**Response to Academic Editor**

**Comment E1: Sampling strategy and selection process for the final 1,276 texts**

*Please clarify the sampling strategy and the selection process for the final 1,276 texts, including inclusion and exclusion criteria, scraping procedures, treatment of missing data or excluded cases, and the role of platform or account-level controls.*

Response: We thank the Editor for this request for greater methodological transparency. Section 3.1 has been substantially restructured to make the selection process fully explicit. The revised section now describes a two-stage sequential funnel:

- Stage 1 (algorithmic cleaning): From the 3,184 texts initially retrieved via keyword-based crawling, exact duplicates were removed by hash comparison, near-duplicates by SimHash (Manku et al., 2007), suspected bot-generated content by account-level flagging, and fixed-template promotional texts by manual spot-checking, yielding 3,184 posts entering Stage 2.
- Stage 2 (line-by-line manual review): Two members of the research team independently reviewed all 3,184 texts. Posts were excluded if (a) the keyword appeared in a non-occupational context, (b) the content was semantically ambiguous or too brief, or (c) the post was clearly unrelated to workplace burnout after reading in full. Disagreements were resolved through discussion. This stage yielded the final corpus of 1,276 posts.

Regarding missing data: among the 1,276 retained posts, 41 lacked one or more metadata fields required for regression (like count, platform, year, or follower count). These 41 posts were included in the discourse analysis but excluded from the regression models, yielding N = 1,235 for Section 4.2. Posts excluded at Stage 2 were not imputed or replaced, as the corpus is purposive rather than probabilistic. This is now explicitly stated in Section 3.1.

Regarding platform and account-level controls: account follower count (log(followers)) is included as a covariate in all regression models to control for account-level exposure differences. Platform is included as a five-category dummy variable (reference = Bilibili). Both are now defined clearly in Section 3.2.

**Comment E2: Define OLS and log regression; provide a brief explanation of the OLS model**

*Please define OLS and log regression clearly and provide a brief explanation of the OLS model.*

Response: Section 3.2 now includes a dedicated paragraph defining Ordinary Least Squares (OLS) regression and the rationale for the log transformation. The formal model specification is also provided:

log(likes + 1) = β0 + β1(sentiment) + β2(platform) + β3(year) + β4(text_len) + β5(keyword) + β6(log(followers)) + ε

The paragraph explains that OLS minimises the sum of squared residuals to estimate linear relationships, that the log(likes + 1) transformation is applied to address the heavy right-skew of raw like counts (mean = 114.3, median = 2, maximum = 34,639, 35.1% zeros), and that the log-transformed specification models proportional rather than absolute changes. All standard errors use the HC1 heteroskedasticity-consistent estimator.

**Comment E3: Justify the use of PCA**

*Please justify the use of PCA for sentiment score fusion.*

Response: A dedicated justification for the use of PCA has been added to Section 3.2, immediately following the technical description of the dual-model setup. Three reasons are now stated explicitly:

- PCA is data-driven and extracts shared variance without imposing a priori weights, reducing researcher-imposed bias in model combination.
- The two models operate on entirely different algorithmic principles and were trained on different corpora, making their outputs only weakly correlated (r = 0.227); PCA's first principal component captures their consensus signal while attenuating each model's idiosyncratic biases.
- The equal loadings of 0.707 on both models confirm genuine equal-weight fusion, and the 61.36% explained variance confirms that a meaningful proportion of shared sentiment information is captured in a single composite score.

**Comment E4: Report robustness analyses more explicitly**

*Please report the robustness analyses more explicitly.*

Response: The robustness check paragraph in Section 4.2 has been revised to be more explicit. A new introductory sentence now frames the purpose of the checks before the results are presented: the analysis assesses whether the primary finding is sensitive to the choice of log transformation, the treatment of zero-like observations, or the distributional assumption. A concluding sentence after Table 4 explicitly states that all three specifications confirm the direction and significance of the finding, ruling out artefact explanations. The three model specifications and their PCA coefficients are reported in full in Table 4 with notes clarifying the non-comparability of raw coefficients across models.

**Comment E5: Ensure consistent description of research design throughout**

*Please ensure that the research design is described consistently throughout the manuscript.*

Response: The research design is now described consistently as a "cross-sectional observational computational social science paradigm" throughout the manuscript. This phrasing appears in the Abstract (Methods section), Section 3.2 (opening sentence), and Section 5.3 (Limitations, third point). The previous inconsistency between "cross-sectional, descriptive, and exploratory" (some locations) and other formulations has been resolved.

**Comment E6: Define all acronyms and methodological terms at first use**

*Please ensure that all acronyms and methodological terms are defined at first use.*

Response: All acronyms have been reviewed and defined at first use. The following were added or corrected:

- **OLS:** now defined as "Ordinary Least Squares (OLS)" at first use in Section 3.2.
- **ICC:** now written as "Intraclass Correlation Coefficient (ICC(2,1))" at first use in Section 3.2.
- **PPML:** "Poisson Pseudo-Maximum Likelihood (PPML)" was already defined at first use and has been retained.
- **PCA:** "principal component analysis (PCA)" was already defined at first use and has been retained.
- **HC1:** "HC1 heteroskedasticity-consistent standard errors" was already explained at first use and has been retained.

**Comment E7: Data Availability Statement and OSF repository**

*Please ensure that the Data Availability Statement and OSF repository provide sufficient materials for verification and replication.*

Response: The Supplementary Materials statement has been updated to explicitly describe the contents of the OSF repository (https://osf.io/ca42g):

- S1: Cleaned corpus (1,276 texts with metadata)
- S2: Open coding table (29 open codes with frequencies and representative text examples)
- S3: Search terms and keywords methodology document (34 keywords with per-keyword post counts and exclusion keyword lists)

This makes it possible for readers to verify the keyword selection, the corpus composition, and the coding framework independently. Analysis scripts are available from the corresponding author upon reasonable request.

**Comment E8: Submit a clean version free of tracked changes, duplicated text, and formatting problems**

*Please submit a clean version of the manuscript free of tracked changes, duplicated text, formatting problems, or residual text from earlier revisions.*

Response: The manuscript has been carefully reviewed for all such issues. Specifically:

- All tracked changes have been accepted and the clean version is submitted as the 'Manuscript' file.
- A duplicated passage in Section 3.1 that described the 3,184-to-1,276 filtering process twice (once in the original text and once in the newly added two-stage funnel paragraph) has been merged into a single coherent account.
- A duplicate description of the log transformation rationale that appeared in both the RQ2 paragraph and the OLS definition paragraph in Section 3.2 has been consolidated into the OLS definition paragraph only.
- A duplicate introductory sentence in the robustness checks paragraph has been removed.
- A repeated report of the human annotation validation statistics (ICC, alpha, kappa) that appeared in both Section 3.2 and Section 4.2 has been resolved: the full statistics remain in Section 3.2 and Section 4.2 now contains a brief cross-reference only.

**Response to Reviewer #1**

We thank Reviewer #1 for the thorough and constructive final-round review. All points raised have been addressed. The responses to the methodological transparency comments (sampling strategy, OLS definition, PCA justification, missing data, robustness analysis, and research design description) are provided in full above under the Editor's comments E1-E8, as these overlap substantially. We address here the additional points specific to Reviewer #1's comments.

**Comment R1: Whether there were any other important control variables**

*Please mention whether there were any other important control variables.*

Response: Section 3.2 now explicitly lists all covariates included in the regression models: platform (5 categories, reference = Bilibili), year (2022-2026, reference = 2022), text length (text_len), source keyword (top 10 keywords as dummy variables, remainder as 'other', reference = exploitation), and account follower count (log(followers)). A sentence has been added noting that follower count is included specifically to control for account-level exposure capacity, which would otherwise confound the sentiment-resonance relationship (the suppression effect described in Section 4.2). No other account-level metadata (e.g., account age, verified status) was systematically available across all five platforms, and this is acknowledged as a limitation in Section 5.3.

**Comment R2: Tone down interpretative statements to avoid causal inferences that exceed the scope of a cross-sectional design**

*Tone down some interpretative statements to avoid causal inferences that exceed the scope of a cross-sectional design, as this could lead to the risk of over-interpretation.*

Response: We thank Reviewer #1 for this important reminder. All interpretative statements in the Discussion have been reviewed and revised to reflect the associational and exploratory nature of the study. The following specific changes were made:

- **Section 5.1, paragraph 1:** "this study finds that workers...have systematically deconstructed this discursive logic" revised to "this study suggests that workers...appear to be engaging in a systematic deconstruction of this discursive logic."
- **Section 5.1, paragraph 1:** "demonstrating that workers not only perceive oppression but have developed a discourse system" revised to "suggesting that workers not only perceive oppression but may have developed a discourse system."
- **Section 5.1, paragraph 3:** "the systemic collapse of work meaning and personal accomplishment becomes a predictable outcome" revised to "is theoretically expected as a predictable outcome (Demerouti et al., 2001)," making clear this is a theoretical proposition rather than an empirical finding of the present study.
- **Section 5.1, paragraph 4:** "C1...is the institutional root cause of burnout...C3...is burnout's direct consequence" revised to "C1...is interpreted as the institutional root cause of burnout...C3...as burnout's direct consequence," signalling that these are theoretical interpretations of the discourse patterns rather than established causal relationships.
- **Section 5.1, paragraph 4:** "Conservation of Resources (COR) theory provides a theoretical explanation for this chain: when workers' core resources...the spiral depletion of resources on the one hand leads through...to identity crisis (C2), and on the other hand directly gives rise to physical exhaustion" revised to "One possible theoretical explanation, drawing on COR theory, is that when workers' core resources...resource depletion may on the one hand contribute to identity crisis through pathways such as frustrated job-seeking and meaning dissolution (C2), and on the other hand be associated with physical exhaustion and micro-resistance practices (C3)."
- **Section 5.2, paragraph 2:** "only a narrative framework that translates individual suffering into structural critique can break through the boundaries of the private sphere" revised to "a narrative framework that translates individual suffering into structural critique appears more likely to break through the boundaries of the private sphere."
- **Section 5.2, paragraph 3:** "completing through a 'like' a miniature 'collective unloading of emotional labour'" revised to "suggesting that a 'like' in this context may function as a miniature 'collective unloading of emotional labour.'"
- **Section 5.2, paragraph 4:** "will systematically misestimate the relationship" revised to "risks systematically misestimating the relationship."

These revisions consistently replace deterministic language with hedged formulations ("may," "appears to," "is interpreted as," "one possible explanation") appropriate to a cross-sectional observational design.

**Comment R3: Reconsider whether the methodological design should be described as 'observational computational social science study' or 'cross-sectional observational computational social science study'**

*Reconsider whether the methodological design should be an 'observational computational social science study' or a 'cross-sectional observational computational social science study'. The widely recognised research paradigms are: positivist, post-positivist, constructivist, critical and pragmatic.*

Response: We thank Reviewer #1 for this clarification. The design is now consistently described as a "cross-sectional observational computational social science" study throughout the manuscript (Abstract, Section 3.2, and Section 5.3), as it is both cross-sectional (data collected at a single time point across platforms) and observational (no experimental manipulation). Regarding the epistemological paradigm: this study most closely aligns with a pragmatic paradigm, in that it integrates qualitative grounded theory coding with quantitative regression modelling to address a practically motivated research question about workplace burnout discourse. This mixed-methods integration does not fit neatly within a single paradigm from the list provided; accordingly, we have not added a specific paradigm label to the text to avoid overstating the philosophical commitment of what is primarily a methodologically descriptive study. We believe the cross-sectional observational framing, combined with the explicit statement that the study is descriptive and exploratory rather than causal, adequately conveys the epistemological boundaries of the work.

**Response to Reviewer #4**

Reviewer #4 has recommended acceptance. We are grateful for the reviewer's positive assessment and their sustained engagement across all rounds of review. No further response is required beyond the revisions described above.

**Summary of Changes in This Round**

The following table summarises all changes made in response to this round of review.

| **Comment** | **Change made** | **Location** |
| --- | --- | --- |
| E1 / R1 | Two-stage funnel description added; missing data treatment stated; 41-post exclusion explained; platform/follower controls clarified | Section 3.1 (revised paragraph) |
| E2 | OLS defined; log transformation rationale explained; formal model equation added | Section 3.2 (new paragraph before robustness checks) |
| E3 | Three-reason justification for PCA added | Section 3.2 (after PCA technical description) |
| E4 | Robustness check paragraph restructured with explicit introductory and concluding sentences | Section 4.2 (robustness paragraph and Table 4 note) |
| E5 | Design description unified as 'cross-sectional observational computational social science paradigm' | Abstract, Section 3.2, Section 5.3 |
| E6 | OLS and ICC defined at first use | Section 3.2 |
| E7 | OSF repository contents explicitly described (S1, S2, S3) | Supplementary Materials statement |
| E8 | Duplicate passages removed (3.1 filtering, 3.2 log transformation, 4.2 robustness intro, 4.2 validation stats); clean version submitted | Sections 3.1, 3.2, 4.2 |
| R1 (control variables) | Full covariate list stated; rationale for follower count noted; limitation of unavailable metadata acknowledged | Section 3.2; Section 5.3 |
| R2 (causal language) | 8 specific phrases revised across Discussion: deterministic verbs replaced with hedged formulations (may, appears to, is interpreted as, one possible explanation) | Sections 5.1 and 5.2 |
| R3 (research paradigm) | 'Cross-sectional observational' consistently applied; pragmatic paradigm noted in response without adding label to text | Section 3.2; Section 5.3 |
| Additional: References | Landis & Koch (1977) and J. Zhang et al. (2022) added to reference list; Atlas/Altaş and Ongore/Öngöre spelling unified | Reference list |

We are confident that the revised manuscript now fully addresses all remaining concerns and meets the standards for publication in PLOS ONE. We remain grateful for the editorial team's and reviewers' sustained and constructive engagement throughout this process.
