# Supplementary material for: Discourse, sentiment, and resonance of workplace burnout on Chinese social media: A computational analysis
Source: PLoS One. 2026 Aug 5;21(8):e0346396. doi: 10.1371/journal.pone.0346396 (PMC13440837; doi:10.1371/journal.pone.0346396)
Supplement: Supplementary file 3 [file pone.0346396.s005.doc]

**Response to Reviewers**

Manuscript title: Discourse, Sentiment, and Resonance of Workplace Burnout on Chinese Social Media: A Computational Analysis

Journal: PLOS ONE

We sincerely thank the Editor and both reviewers for their thorough and constructive feedback. All comments have been carefully considered and addressed in the revised manuscript. Below we provide a point-by-point response to each comment. Changes in the manuscript are indicated by [revised section and line numbers where applicable].

We must acknowledge that Generative AI is used to translate human-written Chinese into more readable English in this letter.

**Response to Editor's Additional Comments and Reviewer #4 — Comment #1 (Generalisability of Claims)**

Comment summary: The manuscript moves too quickly from a purposive corpus of digitally active social media users to claims about "Chinese workers," "regions," and "occupational burnout" more generally. All general claims should be revised to refer to the analysed posts or users, and per-platform and per-province sample counts with uncertainty indicators should be provided before retaining any spatial interpretation.

**Response:** We thank the Editor and Reviewer #4 for this important methodological concern. We have made two substantive revisions in response.

First, we have systematically reviewed the entire manuscript and revised all overgeneralised claims. All findings are now consistently framed as patterns observed among "users who actively produce burnout discourse in Chinese digital public spaces," rather than as representative estimates of Chinese workers in general. This framing is explicitly stated in the Data Sources section.

Second, the spatial/regional analysis has been removed entirely from the revised manuscript. Given the small and uneven per-province sample sizes inherent to our purposive corpus, we concluded that tertile-based regional sentiment categories could not be adequately supported by the data and would risk creating a misleading impression of regional burnout differences. This decision is consistent with the Editor's guidance.

**Response to Editor's Comment #E2 and Reviewer #4 — Comment #5 (Resonance Analysis and Causal Language)**

Comment summary: Likes cannot be treated as direct evidence of collective identification, as they are shaped by platform algorithms, posting time, account visibility, text length, and user demographics. The regression did not control for major engagement confounders. Causal language such as "trigger" and "mobilisation" is too strong.

**Response:** We thank the Editor and Reviewer #4 for this important methodological critique. We have made three substantive revisions.

First, we have re-estimated all regression models with an expanded set of covariates including platform (5 categories, reference = Bilibili), year (2022–2026, reference = 2022), text length, source keyword (top 10 dummy variables), and — critically — log(followers) as a proxy for account-level exposure. The inclusion of followers revealed a suppression effect: sentiment coefficients were non-significant across all model specifications without followers (p > 0.45), but became highly significant and consistently negative once follower count was controlled (PCA: β = -0.403, SE = 0.032, p < 0.001). This finding is now reported in full in Section 4.2 and interpreted as reflecting two opposing pathways — an exposure pathway and a resonance pathway — that cancel each other out when account exposure is not held constant.

Second, we note that the original ZINB model failed to converge due to a non-invertible Hessian matrix. The revised analysis uses OLS regression on log(likes+1) as the primary model with HC1 heteroskedasticity-robust standard errors, with Poisson pseudo-maximum likelihood (PPML) and threshold OLS (likes > 0 subsample, n = 849) as robustness checks. Results are consistent across all three specifications (see Table 3).

Third, we have replaced all causal language throughout the manuscript. Terms such as "trigger," "mobilise," and "collective mobilisation" have been replaced with explicitly associational language, including "is associated with," "correlates with," and "suggests a pattern of." Likes are now consistently described as "a proxy for online resonance or engagement" rather than as direct evidence of collective identification.

**Response to Reviewer #1 — Comment #1 (Abstract Structure)**

Comment summary: The abstract should include background, objective, methods, results, and conclusion. Objectives should be stated using non-finite verbs rather than as questions. The methods section should briefly describe research type, design, sample, research techniques and instruments, and data analysis.

**Response:** We thank Reviewer #1 for this structural feedback. The abstract has been fully rewritten according to the five-component structure (background — objectives — methods — results — conclusions). Specifically: (1) the background now contextualises occupational burnout as a systemic organisational challenge and positions social media as an underutilised data source; (2) objectives are now stated as declarative purpose statements using non-finite verbs ("to investigate," "to examine"), not as questions; (3) the methods component now specifies the research type (computational social science, cross-sectional exploratory design), sample (n = 1,276 texts from five platforms), techniques (grounded theory coding, dual-model PCA sentiment fusion, OLS and PPML regression), and key validation indicators (Fleiss' Kappa = 0.792; ICC(2,1) = 0.870); (4) results report the main findings from both RQ1 and RQ2 with specific statistics; and (5) the conclusion states the theoretical and practical contributions.

**Response to Reviewer #1 — Comment #2 (Introduction Structure)**

Comment summary: The logical coherence of the introduction should be reviewed, and the following elements should be included: (a) general context of the problem, (b) digital transformation and social media as a data source, (c) theoretical and conceptual approaches, (d) computational approach, (e) state of the art, (f) knowledge gap, (g) contribution and objectives.

**Response:** We thank Reviewer #1 for this detailed structural guidance. The introduction has been reviewed and reorganised to ensure all seven elements are present and logically sequenced. Specifically: (a) the opening paragraph establishes the global prevalence and organisational stakes of burnout; (b) the second paragraph positions social media as a timely and contextually appropriate data source, noting the limitations of traditional survey methods; (c) the third paragraph introduces the diachronic discursive shift in Chinese workplace culture as theoretical background, and the theoretical section explicitly integrates emotional labour and affective publics frameworks; (d) the analytical approach paragraph describes the computational pipeline (grounded theory coding, dual-model sentiment fusion, regression modelling); (e) relevant prior computational studies on burnout discourse and social media sentiment are cited throughout; (f) the gap is explicitly stated: existing research relies predominantly on structured scales and cannot capture the spontaneous, interactive, and structurally embedded nature of burnout expression in digital public spaces; (g) the contribution and the two research questions are stated clearly at the close of the introduction.

**Response to Reviewer #1 — Comment #3 and Reviewer #4 — Comment #2 (Methods: Design Statement, Reproducibility, and Ethics)**

Comment summary: (1) The stated addition of a "computational social science / cross-sectional, descriptive, exploratory design" declaration was not found in Section 3.2 (Line 272) of the submitted manuscript. (2) Reproducibility is insufficient: exact crawling/API procedures, query dates, search strings, inclusion/exclusion counts, SimHash and bot-detection thresholds, code, model/package versions, and random seeds are needed. The inconsistency between the submission form marking ethics as "N/A" and the manuscript reporting full IRB approval should also be resolved.

**Response:** We thank both reviewers for identifying these gaps.

Regarding the design declaration: Section 3.2 now opens with an explicit statement: "This study follows a computational social science paradigm and adopts a cross-sectional, descriptive, and exploratory design. The research questions focus on descriptive exploration and theoretical interpretation of discourse and resonance patterns, rather than causal inference."

Regarding reproducibility, the following information has been added to the Methods section and Supplementary Materials: (1) data collection date: 15 January 2026; (2) the complete list of 34 search terms is provided in Supplementary Material S1; (3) the exclusion keyword lists (74 terms) used to filter fan culture and entertainment content are documented in Supplementary Material S3; (4) the filtering pipeline is described step by step, including hash-based deduplication, SimHash near-duplicate detection, and manual line-by-line review of the full corpus (n = 3,184 → n = 1,276 after manual review); (5) the RoBERTa model is identified as IDEA-CCNL/Erlangshen-RoBERTa-110M-Sentiment; (6) all package versions are reported (Python 3.14; transformers 5.10.1; torch 2.12.0; snownlp 0.12.3; scikit-learn 1.8.0; statsmodels 0.14.6; pandas 3.0.1; numpy 2.4.3); (7) Given that we have replaced the machine learning text analysis approach with manual line-by-line coding for grounded theory, the concern regarding random seeds is no longer applicable.

Regarding the ethics inconsistency: the submission form has been corrected. The manuscript reports IRB approval (approval number THU-EC-AICB003), and the submission form now reflects this consistently.

**Response to Reviewer #1 — Comment #4 (Results Structure)**

Comment summary: The results should be presented in accordance with the research objectives. The study objectives can be inferred from the proposed research questions.

**Response:** We thank Reviewer #1 for this comment. The Results section has been restructured to explicitly correspond to the two research questions. Section 4.1 (Discourse Analysis) directly addresses RQ1 by reporting the grounded theory coding outcomes — 29 open codes, 6 axial themes, and 3 core categories — and interpreting their theoretical significance. Section 4.2 (Sentiment and Resonance Analysis) directly addresses RQ2 by reporting the sentiment descriptive statistics, the suppression effect of follower count, the full regression model, and the robustness checks. Each subsection opens with a sentence explicitly linking the findings to the corresponding research question, ensuring the reader can trace results back to the stated objectives throughout.

**Response to Reviewer #1 — Comment #5 (Discussion Structure)**

Comment summary: Figures should not be included in the discussion; they should be included in the results section. The discussion should interpret the results with critical analysis considering the theoretical and conceptual framework and comparing them with previous studies. Theoretical and practical implications and limitations should also be included.

**Response:** We thank Reviewer #1 for this comment. Three revisions have been made.

First, all figures and tables have been relocated to the Results section. No figures or tables appear in the Discussion.

Second, the Discussion has been substantially expanded to provide critical interpretation of findings against the theoretical frameworks. Section 5.1 interprets the discourse structure through emotional labour theory (Hochschild, 2012; Jaikla & Piyakun, 2024) and JD-R model (Demerouti et al., 2001), and explicitly compares findings with prior studies on Chinese workplace discourse (Yang & Jiao, 2024; Wang, 2020; Liu & Chen, 2025; Matthyssen, 2024). Section 5.2 interprets the resonance findings through affective publics theory (Papacharissi, 2016; Dai, 2025) and connects the suppression effect finding to methodological debates in social media research (Fernández-Gavilanes et al., 2019).

Third, a dedicated Limitations and Future Directions subsection (Section 5.3) has been added, addressing: (1) platform mediation and representativeness constraints; (2) the limitations of likes as a resonance proxy; and (3) the cross-sectional design's inability to support causal inference. Practical implications are stated in a separate paragraph at the close of the Discussion, recommending that organisations treat high-resonance structural critique discourse as an early warning signal warranting systemic rather than individual-level intervention.

**Response to Reviewer #1 — Comment #6 (Conclusions)**

Comment summary: The conclusions should be summarised in response to the research objectives, and should address contributions, implications, and scope.

**Response:** We thank Reviewer #1 for this guidance. Due to space constraints, we have integrated the conclusions into Section 5.3. Since the theoretical contributions are already explicitly addressed in Sections 5.1 and 5.2, Section 5.3 now opens directly with the practical implications.

**Response to Reviewer #4 — Comment #3 (Topic Modelling Pipeline)**

Comment summary: The pipeline depends on exploratory BERTopic, manual theme naming, and subsequent LLM classification, while the "Others" category still contains 35.38% of the corpus. A stability check, topic coherence evidence, and a human-versus-LLM confusion matrix for the validation subset are needed.

**Response:** We thank Reviewer #4 for this substantive methodological critique. This comment prompted a fundamental revision of the thematic analysis approach.

In the revised manuscript, the BERTopic + LLM classification pipeline has been replaced entirely with a grounded theory three-layer coding procedure (Strauss & Corbin, 1998). All themes now emerge inductively from the data without any pre-specified framework. The revised procedure is as follows: (1) open coding — three independent coders read all 1,276 texts line by line, extracting 29 initial conceptual labels; (2) axial coding — codes were clustered into 6 higher-order themes based on logical relationships; (3) selective coding — three core categories were abstracted. Inter-rater reliability was assessed using Fleiss' Kappa (κ = 0.792, Substantial agreement; Landis & Koch, 1977), with pairwise Cohen's Kappa values ranging from 0.704 to 0.842. Disagreements were resolved through group discussion.

This approach directly resolves the concerns raised: the "Others" category no longer exists, as every text is assigned a primary axial code; stability and coherence are demonstrated through the Kappa statistics and the saturation criterion (no new concepts emerged after approximately the 1,000th text); and the inter-rater agreement metrics replace the previously inadequate human-vs-LLM validation. The complete open coding table with frequencies and representative text examples is provided in Supplementary Material S2.

**Response to Reviewer #4 — Comment #4 (Sentiment Analysis Validation)**

Comment summary: The manuscript reports targeted positive-class accuracy but not full performance over sentiment classes. The exact RoBERTa model, the mapping from classifier output to the [-1, 1] sentiment score, class distribution, inter-annotator agreement, confusion matrix, macro-F1, and a sensitivity analysis for ironic cases are needed.

**Response:** We thank Reviewer #4 for this detailed methodological request. The sentiment validation has been substantially expanded in the revised manuscript and three columns of numerical values are listed in Supplementary Material S1..

The following information is now reported: (1) the exact model is IDEA-CCNL/Erlangshen-RoBERTa-110M-Sentiment; (2) the score mapping is P(Positive) − P(Negative), yielding a continuous score in [−1, 1]; (3) sentiment class distributions are reported for all three measures (RoBERTa: 54.8% negative, 8.4% neutral, 36.8% positive; SnowNLP: 38.5% / 13.2% / 48.3%; PCA fusion: 43.9% / 16.1% / 40.0%); (4) rather than relying on a single model as ground truth, validation was conducted against human annotations: 300 texts were stratified-sampled by PCA score and independently rated by three trained annotators on a 5-point Likert scale; inter-rater reliability was high (ICC(2,1) = 0.870, Excellent; Krippendorff's α = 0.935; weighted κ mean = 0.772, Substantial); agreement between PCA scores and human consensus ratings was 85.3%; (5) A cross-model confusion matrix between RoBERTa and SnowNLP classifications is reported (N = 1,276; classification threshold = ±0.2). The two models achieved an overall agreement rate of 55.0% (702/1,276). Class-level performance was as follows: negative class F1 = 0.601 (Precision = 0.537, Recall = 0.683); positive class F1 = 0.547 (Precision = 0.569, Recall = 0.526); neutral class F1 = 0.000, as RoBERTa produced no neutral predictions under the ±0.2 threshold. The overall Macro-F1 = 0.383, reflecting moderate inter-model divergence attributable to systematic algorithmic differences — RoBERTa (Transformer-based, trained on diverse Chinese corpora) is more sensitive to negative semantic content, while SnowNLP (Naïve Bayes, trained on product reviews) tends toward positive classifications — rather than indicating error in either model. This divergence is precisely why PCA fusion was adopted: to extract the shared signal between two independently biased estimators and yield a more conservative composite measure; (6) an irony sensitivity analysis was conducted using a 19-term detection lexicon, identifying 147 potentially ironic texts (4.7%); all three sentiment measures showed higher rates of positive classification among ironic texts (59.9%–70.1%) compared to non-ironic texts (35.6%–47.3%), and this caveat is noted in the Limitations section.

**Response to Reviewer #4 — Comment #6 (Related Work: Metadata-Aware Sentiment Modelling)**

Comment summary: The computational sentiment component should be better positioned against recent studies on social media sentiment and metadata-aware modelling, for example "Enhancing Sentiment Analysis on Social Media: Integrating Text and Metadata for Refined Insights" (2024).

**Response:** We thank Reviewer #4 for this suggestion. The related work section and the Methods section have been revised to engage with this literature. Guerrero Contreras et al. (2024) is now cited in the Methods section to contextualise our decision to incorporate metadata covariates (platform, year, text length, follower count) into the regression models, and to explain why our approach relies primarily on text-based sentiment scores rather than metadata-augmented sentiment features: our primary analytical goal is to examine the relationship between sentiment polarity and resonance while controlling for confounders, rather than to maximise sentiment classification accuracy per se. The full reference has been added to the reference list. This reference is already present in the current version of the manuscript (Guerrero Contreras et al., 2024, cited in Section 3.2).

**Response to Reviewer #4 — Comment #7 (Title Inconsistency)**

Comment summary: The title is inconsistent across the submission metadata and the manuscript body, where "Discourse, Sentiment, and Resonance of Workplace Burnout on Chinese Social Media: A Computational Analysis" and "What Burns You Out? Discourse, Sentiment and Resonance in Chinese Workplace" both appear. One final title should be used consistently throughout all submission files.

**Response:** We thank Reviewer #4 for identifying this inconsistency. The title has been unified across all submission files as:

Discourse, Sentiment, and Resonance of Workplace Burnout on Chinese Social Media: A Computational Analysis

The alternative title ("What Burns You Out?...") was the previous version (R0).

**Response to Editor's Comment #E4 and Reviewer #4 — Comment #8 (Rhetorical Language and Terminology)**

Comment summary: (1) Strong rhetorical expressions such as "collective mobilisation," "emotional sovereignty," and "high-conductivity material" should be replaced with more empirically grounded and methodologically neutral language. (2) Grammatical and spelling errors should be corrected, including "means wordking," inconsistent capitalisation of RoBERTa. (3) Overtly definitive labels such as "low occupational burnout" for tertile-based sentiment categories should be replaced with neutral wording.

**Response:** We thank the Editor and Reviewer #4 for this careful attention to language. Three sets of revisions have been made.

First, all rhetorically strong expressions have been replaced throughout the manuscript. "Collective mobilisation" is now rendered as "patterns of collective online engagement"; "emotional sovereignty" has been removed entirely; "high-conductivity material" has been replaced with "a discourse frame that more readily elicits collective resonance." All remaining instances of causal language ("trigger," "mobilise," "drive") in the context of likes and sentiment have been replaced with associational language ("is associated with," "correlates with," "is observed alongside").

Second, all identified typographical and grammatical errors have been corrected. "Means wordking" has been corrected to "meaning working." RoBERTa capitalisation has been standardised to "RoBERTa" throughout the manuscript, including in tables and figure captions.

Third, the spatial analysis based on tertile-based sentiment categories has been removed from the manuscript entirely (see also our response to Comment #1/#E3). This removes the problematic labels "low occupational burnout," "medium occupational burnout," and "high-risk occupational burnout" that were attached to tertile groupings the data could not adequately support.

**Response to Reviewer #4 — Comment #9 (Figure and Table Captions)**

Comment summary: Figures 2–4 and Table 2 need more self-contained captions. Captions should state the sample size, excluded observations, model specification, baseline category, covariates, and whether reported margins are derived from the ZINB count component.

**Response:** We thank Reviewer #4 for this comment. All table and figure captions in the revised manuscript have been expanded to be fully self-contained. Specifically, each caption now includes: (1) the analytic sample size and number of excluded observations where applicable; (2) the model specification (e.g., OLS with HC1 robust standard errors, Poisson PPML); (3) the reference/baseline category for all categorical variables (platform reference = Bilibili; year reference = 2022; keyword reference = "exploitation/压榨"); (4) the full list of covariates included in the model; and (5) clarification of the dependent variable transformation (log(likes+1) for OLS models; raw like counts for Poisson PPML and threshold OLS).

Regarding the ZINB count component: As noted in our response to Comment #5, the ZINB model has been abandoned. This decision was made because we conducted a comprehensive manual coding of the entire dataset and applied more rigorous sample filtering. When this refined dataset was input into the ZINB model, it failed to converge (due to a non-invertible Hessian matrix). The revised analysis uses OLS, Poisson PPML, and threshold OLS as the three model specifications, and all captions reflect this change. The question of which component margins are derived from is therefore no longer applicable, and the captions note the model type and dependent variable accordingly for each table.

We believe the revised manuscript is substantially strengthened by these revisions and hope it now meets the standards for publication in PLOS ONE. We remain grateful for the reviewers' and editor's detailed and constructive engagement with our work.
