# Supplementary material for: Discourse, sentiment, and resonance of workplace burnout on Chinese social media: A computational analysis
Source: PLoS One. 2026 Aug 5;21(8):e0346396. doi: 10.1371/journal.pone.0346396 (PMC13440837; doi:10.1371/journal.pone.0346396)
Supplement: Peer review history

# Peer review: Decision Letter 0

**DOI:** 10.1371/journal.pone.0346396.r001

**Authors:** Jenna Scaramanga

5 Apr 2026

PONE-D-26-05195

What Burns You Out? Discourse, Sentiment and Resonance in Chinese Workplace

PLOS One

Dear Dr. Yin,

Thank you for submitting your manuscript to PLOS ONE. After careful consideration, we feel that it has merit but does not fully meet PLOS ONE’s publication criteria as it currently stands. Therefore, we invite you to submit a revised version of the manuscript that addresses the points raised during the review process.

Your submission has been evaluated by three reviewers, and their comments are appended below. Please carefully revise your manuscript to address the points raised.

Please submit your revised manuscript by May 17 2026 11:59PM. If you will need more time than this to complete your revisions, please reply to this message or contact the journal office at plosone@plos.org. When you're ready to submit your revision, log on to https://www.editorialmanager.com/pone/ and select the 'Submissions Needing Revision' folder to locate your manuscript file.

Please include the following items when submitting your revised manuscript:

- A letter that responds to each point raised by the academic editor and reviewer(s). You should upload this letter as a separate file labeled 'Response to Reviewers'.
- A marked-up copy of your manuscript that highlights changes made to the original version. You should upload this as a separate file labeled 'Revised Manuscript with Track Changes'.
- An unmarked version of your revised paper without tracked changes. You should upload this as a separate file labeled 'Manuscript'.

If you would like to make changes to your financial disclosure, please include your updated statement in your cover letter. Guidelines for resubmitting your figure files are available below the reviewer comments at the end of this letter.

If applicable, we recommend that you deposit your laboratory protocols in protocols.io to enhance the reproducibility of your results. Protocols.io assigns your protocol its own identifier (DOI) so that it can be cited independently in the future. For instructions see: https://journals.plos.org/plosone/s/submission-guidelines#loc-laboratory-protocols. Additionally, PLOS ONE offers an option for publishing peer-reviewed Lab Protocol articles, which describe protocols hosted on protocols.io. Read more information on sharing protocols at https://plos.org/protocols?utm_medium=editorial-email&utm_source=authorletters&utm_campaign=protocols.

As the corresponding author, your ORCID iD is verified in the submission system and will appear in the published article. PLOS supports the use of ORCID, and we encourage all coauthors to register for an ORCID iD and use it as well. Please encourage your coauthors to verify their ORCID iD within the submission system before final acceptance, as unverified ORCID iDs will not appear in the published article. *Only*  the individual author can complete the verification step; PLOS staff *cannot*  verify ORCID iDs on behalf of authors.

We look forward to receiving your revised manuscript.

Kind regards,

Jenna Scaramanga

Staff Editor

PLOS One

Journal Requirements:

When submitting your revision, we need you to address these additional requirements.

1. Please ensure that your manuscript meets PLOS ONE's style requirements, including those for file naming. The PLOS ONE style templates can be found at

https://journals.plos.org/plosone/s/file?id=wjVg/PLOSOne_formatting_sample_main_body.pdf and

https://journals.plos.org/plosone/s/file?id=ba62/PLOSOne_formatting_sample_title_authors_affiliations.pdf

2. In your Methods section, please include additional information about your dataset and ensure that you have included a statement specifying whether the collection and analysis method complied with the terms and conditions for the source of the data.

3. Please note that PLOS One has specific guidelines on code sharing for submissions in which author-generated code underpins the findings in the manuscript. In these cases, we expect all author-generated code to be made available without restrictions upon publication of the work. Please review our guidelines at https://journals.plos.org/plosone/s/materials-and-software-sharing#loc-sharing-code and ensure that your code is shared in a way that follows best practice and facilitates reproducibility and reuse.

4. We note that Figure 1 and 3 in your submission contain [map/satellite] images which may be copyrighted. All PLOS content is published under the Creative Commons Attribution License (CC BY 4.0), which means that the manuscript, images, and Supporting Information files will be freely available online, and any third party is permitted to access, download, copy, distribute, and use these materials in any way, even commercially, with proper attribution. For these reasons, we cannot publish previously copyrighted maps or satellite images created using proprietary data, such as Google software (Google Maps, Street View, and Earth). For more information, see our copyright guidelines: http://journals.plos.org/plosone/s/licenses-and-copyright.

We require you to either (1) present written permission from the copyright holder to publish these figures specifically under the CC BY 4.0 license, or (2) remove the figures from your submission:

1. You may seek permission from the original copyright holder of Figure 1 and 3 to publish the content specifically under the CC BY 4.0 license.

We recommend that you contact the original copyright holder with the Content Permission Form (http://journals.plos.org/plosone/s/file?id=7c09/content-permission-form.pdf) and the following text:

“I request permission for the open-access journal PLOS ONE to publish XXX under the Creative Commons Attribution License (CCAL) CC BY 4.0 (http://creativecommons.org/licenses/by/4.0/). Please be aware that this license allows unrestricted use and distribution, even commercially, by third parties. Please reply and provide explicit written permission to publish XXX under a CC BY license and complete the attached form.”

Please upload the completed Content Permission Form or other proof of granted permissions as an ""Other"" file with your submission.

In the figure caption of the copyrighted figure, please include the following text: “Reprinted from [ref] under a CC BY license, with permission from [name of publisher], original copyright [original copyright year].”

2. If you are unable to obtain permission from the original copyright holder to publish these figures under the CC BY 4.0 license or if the copyright holder’s requirements are incompatible with the CC BY 4.0 license, please either i) remove the figure or ii) supply a replacement figure that complies with the CC BY 4.0 license. Please check copyright information on all replacement figures and update the figure caption with source information. If applicable, please specify in the figure caption text when a figure is similar but not identical to the original image and is therefore for illustrative purposes only.

The following resources for replacing copyrighted map figures may be helpful:

USGS National Map Viewer (public domain): http://viewer.nationalmap.gov/viewer/

The Gateway to Astronaut Photography of Earth (public domain): http://eol.jsc.nasa.gov/sseop/clickmap/

Maps at the CIA (public domain): https://www.cia.gov/library/publications/the-world-factbook/index.html and https://www.cia.gov/library/publications/cia-maps-publications/index.html

NASA Earth Observatory (public domain): http://earthobservatory.nasa.gov/

Landsat: http://landsat.visibleearth.nasa.gov/

USGS EROS (Earth Resources Observatory and Science (EROS) Center) (public domain): http://eros.usgs.gov/#

Natural Earth (public domain): http://www.naturalearthdata.com

5. If the reviewer comments include a recommendation to cite specific previously published works, please review and evaluate these publications to determine whether they are relevant and should be cited. There is no requirement to cite these works unless the editor has indicated otherwise.

[Note: HTML markup is below. Please do not edit.]

Reviewers' comments:

Reviewer's Responses to Questions

**Comments to the Author**

1. Is the manuscript technically sound, and do the data support the conclusions?

The manuscript must describe a technically sound piece of scientific research with data that supports the conclusions. Experiments must have been conducted rigorously, with appropriate controls, replication, and sample sizes. The conclusions must be drawn appropriately based on the data presented.

Reviewer #1: Partly

Reviewer #2: No

Reviewer #3: Partly

**********

2. Has the statistical analysis been performed appropriately and rigorously?

Reviewer #1: Yes

Reviewer #2: Yes

Reviewer #3: No

**********

3. Have the authors made all data underlying the findings in their manuscript fully available?

The PLOS Data policy requires authors to make all data underlying the findings described in their manuscript fully available without restriction, with rare exception (please refer to the Data Availability Statement in the manuscript PDF file). The data should be provided as part of the manuscript or its supporting information, or deposited to a public repository. For example, in addition to summary statistics, the data points behind means, medians and variance measures should be available. If there are restrictions on publicly sharing data—e.g. participant privacy or use of data from a third party—those must be specified.

Reviewer #1: No

Reviewer #2: No

Reviewer #3: Yes

**********

4. Is the manuscript presented in an intelligible fashion and written in standard English?

PLOS ONE does not copyedit accepted manuscripts, so the language in submitted articles must be clear, correct, and unambiguous. Any typographical or grammatical errors should be corrected at revision, so please note any specific errors here.

Reviewer #1: No

Reviewer #2: Yes

Reviewer #3: No

**********

5. Review Comments to the Author

Please use the space provided to explain your answers to the questions above. You may also include additional comments for the author, including concerns about dual publication, research ethics, or publication ethics. (Please upload your review as an attachment if it exceeds 20,000 characters)

Reviewer #1: Dear Authors, on behalf of PLOS ONE, we thank you for submitting your manuscript, “What Burns You Out? Discourse, Sentiment, and Resonance in the Chinese Workplace.” We note that this is an original, innovative, and interdisciplinary study combining Natural Language Processing with organizational psychology. Furthermore, the overall similarity percentage is 16%, and the percentage of text generated by AI is 69%. As required by PLOS ONE (https://journals.plos.org/plosone/s/submission-guidelines#loc-abstract), the manuscript’s structure must follow the IMRDC format. To evaluate the manuscript, we have used the MMAT guidelines (http://mixedmethodsappraisaltoolpublic.pbworks.com/w/file/fetch/127916259/MMAT__criteria-manual_2018-08-01_ENG.pdf) for exploratory mixed-methods studies. In accordance with these guidelines, we suggest improving the following methodological aspects: 1) Rewrite the abstract to include a brief description of the problem/theory, objectives, methods, key results, and conclusions. 2) The introduction should include a brief, solid theoretical basis and justification. 3) The methods and materials section should include the type and design of the research, unit of analysis, how the sample was selected, eligibility criteria, variables analyzed, data collection period, data extraction procedures, and the methods used for the analysis of quantitative and qualitative data. This section must be clear and precise to ensure reproducibility. 4) Regarding ethical considerations, there is no mention of whether the Ethics Committee that approved the research protocol issued an exemption from the requirement for informed consent, given that publicly available data from the internet was used; nor is there any mention of which data protection policy was applied. 5) Specify the following: how methodological triangulation was performed between qualitative, computational, and theoretical data. Transparency in data preprocessing. How the validity of sentiment measurement and analytical coherence was determined, ensuring the reliability of the results and the study’s reproducibility. 6) Describe the pipeline. Present the structured results (discursive, emotional, and resonance levels) with examples of quotes. Specify which software and methods were used for the qualitative analysis of discourse and sentiment. Specify the version of the ROBERTa model; the techniques for feedback analysis (resonance); the metrics and other statistical methods used. 7) The data and materials available at https://osf.io/eu6mp/files/xeagu are incomplete; the initial data, data from the NLP processing pipeline, and model output data must be presented.

Reviewer #2: The manuscript presents several strengths. The dataset is relatively substantial for studies of online discourse, and the integration of multiple analytical techniques allows the authors to approach the phenomenon from complementary perspectives. In particular, the attempt to link computational findings with sociological interpretations of emotional labor and collective affect provides an interesting analytical lens. The results identifying recurring themes in burnout discourse and exploring the relationship between emotional tone and user engagement contribute to the understanding of how workplace dissatisfaction is articulated and circulated in digital environments.

At the same time, several aspects of the manuscript could be further strengthened in order to enhance its analytical clarity and methodological transparency. First, the theoretical integration between occupational burnout, emotional labor, and affective publics could be articulated more clearly. While the literature review introduces these frameworks individually, the manuscript would benefit from a more explicit explanation of how they jointly inform the research questions and analytical strategy. Clarifying the conceptual pathway that connects individual emotional experiences, digital discourse, and collective resonance would help strengthen the coherence of the theoretical contribution.

Second, the methodological section would benefit from additional detail regarding the data collection and preprocessing procedures. For example, providing clearer information about the keyword selection process, inclusion and exclusion criteria for posts, handling of duplicate or automated content, and the overall sampling logic would improve reproducibility. Given that the study relies heavily on automated text classification and sentiment analysis, a more detailed explanation of the validation procedures used to assess the reliability of the classification results would also be valuable.

Third, some interpretations presented in the discussion appear to extend beyond what can be directly supported by the empirical findings. While the interpretations regarding themes such as “institutional absurdity” and “internal friction resistance” are insightful, presenting these interpretations with slightly greater caution and clearly distinguishing between empirical evidence and theoretical inference would strengthen the analytical balance of the manuscript. This is particularly relevant in the discussion of geographic variation, where the proposed explanations relating to economic transitions and cultural patterns may be plausible but would likely require additional empirical evidence to be fully substantiated.

Finally, the manuscript could benefit from a more developed reflection on the limitations associated with social media data. Although the authors briefly acknowledge that the dataset primarily represents younger and digitally active populations, further discussion of potential biases related to platform dynamics, self-selection of users, and algorithmic amplification would provide useful context for interpreting the findings. Expanding this section would help situate the results within the broader methodological challenges of digital trace data.

These observations should be understood as suggestions aimed at strengthening the manuscript rather than fundamental concerns regarding the relevance of the research. With clearer articulation of the theoretical framework, additional methodological transparency, and a more explicit discussion of the study’s limitations, the manuscript has the potential to make a meaningful contribution to interdisciplinary research on occupational burnout and digital public discourse.

Overall, the study addresses an important topic and presents an innovative analytical approach. I encourage the authors to further develop the conceptual framing and methodological description in order to enhance the clarity and robustness of the manuscript.

Reviewer #3: The article addresses a topic that is interesting from a scientific perspective and may serve as a source of inspiration for researchers; however, both the analysis itself and the interpretation of its results require significant revisions and additions.

1. The title does not accurately reflect the data analysis and the results obtained.

2. What was the purpose of including a subsection on emotional labor in the section on the literature review and theoretical framework, given that this issue was omitted from the analysis and discussion?

3. Data and Sampling Limitations: The sample of 3,691 social media posts may not reflect the broader population of workers in China (overrepresentation of younger, urban, digitally active users). Results may depend on the specific platform (e.g., censorship, algorithms promoting certain types of content). Individuals experiencing stronger burnout are more likely to post, potentially inflating negative sentiment.

4. Issues with Concept Operationalization: (“internal friction resistance” or “institutional absurdity”). Risk of overinterpreting colloquial language as coherent theoretical categories.

5. Limitations of NLP Methods: (BERTopic-depend on model parameters and may be unstable). RoBERTa (sentiment analysis- struggle with: irony, sarcasm ect.), LLM-assisted coding: limited transparency in the coding process)

6. Lack of Methodological Triangulation (Reliance solely on social media text: no interviews, qualitative validation, or organizational data.

7. Causality Issues; The study is descriptive and exploratory, cannot establish what actually causes burnout. Lack of deeper causal analysis in the dynamics of emotions and resonance. The conclusion describes these phenomena but does not sufficiently explore their causes in the context of the collected data. For example, why does “institutional absurdity” resonate the most? The study points to “shared recognition and ironic deconstruction of systemic irrational logic,” but do the textual data actually allow for such a clear interpretation of motivation? Is this the only or primary cause?

8. Spatial Interpretation Concerns“emotional lowland” and “relief zone” oversimplification, does not control for confounding factors (e.g., platform usage, age, language differences, industry distribution).

9. Political and Cultural Context. In China censorship and self-censorship may distort the data, users may rely on metaphor or irony, that models misinterpret. Lack of cultural context in interpreting “emotional exhaustion”: There is insufficient context regarding what “traditional individualized narratives” mean within Chinese culture. Did “emotional exhaustion” carry specific cultural connotations that are now changing? Without this context, the claim of a “shift from passive endurance to active boundary-setting” may be difficult to fully interpret for readers unfamiliar with the nuances of the Chinese work environment.

**********

6. PLOS authors have the option to publish the peer review history of their article (what does this mean?). If published, this will include your full peer review and any attached files.

If you choose “no”, your identity will remain anonymous but your review may still be made public.

**Do you want your identity to be public for this peer review?**  For information about this choice, including consent withdrawal, please see our Privacy Policy.

Reviewer #1: No

Reviewer #2: **Yes:** Alberto Durán González

Reviewer #3: No

**********

[NOTE: If reviewer comments were submitted as an attachment file, they will be attached to this email and accessible via the submission site. Please log into your account, locate the manuscript record, and check for the action link "View Attachments". If this link does not appear, there are no attachment files.]

To ensure your figures meet our technical requirements, please review our figure guidelines: https://journals.plos.org/plosone/s/figures

You may also use PLOS’s free figure tool, NAAS, to help you prepare publication quality figures: https://journals.plos.org/plosone/s/figures#loc-tools-for-figure-preparation.

NAAS will assess whether your figures meet our technical requirements by comparing each figure against our figure specifications.

# Author comment: Author response to Decision Letter 1

**DOI:** 10.1371/journal.pone.0346396.r002

15 Apr 2026

Thank you to the reviewers for taking the time to provide constructive suggestions. We have addressed all the issues point by point, as detailed in the uploaded response letter.

Attachment Submitted filename: response_letter_EN.docx

# Peer review: Decision Letter 1

**DOI:** 10.1371/journal.pone.0346396.r003

**Authors:** Chunyu Zhang

9 Jun 2026

PONE-D-26-05195R1

Discourse, Sentiment, and Resonance of Workplace Burnout on Chinese Social Media: A Computational Analysis

PLOS One

Dear Dr. Yin,

Thank you for submitting your manuscript to PLOS ONE. After careful consideration, we feel that it has merit but does not fully meet PLOS ONE’s publication criteria as it currently stands. Therefore, we invite you to submit a revised version of the manuscript that addresses the points raised during the review process.

Please submit your revised manuscript by Jul 24 2026 11:59PM. If you will need more time than this to complete your revisions, please reply to this message or contact the journal office at plosone@plos.org. When you're ready to submit your revision, log on to https://www.editorialmanager.com/pone/ and select the 'Submissions Needing Revision' folder to locate your manuscript file.

Please include the following items when submitting your revised manuscript:

- A letter that responds to each point raised by the academic editor and reviewer(s). You should upload this letter as a separate file labeled 'Response to Reviewers'.
- A marked-up copy of your manuscript that highlights changes made to the original version. You should upload this as a separate file labeled 'Revised Manuscript with Track Changes'.
- An unmarked version of your revised paper without tracked changes. You should upload this as a separate file labeled 'Manuscript'.

If you would like to make changes to your financial disclosure, please include your updated statement in your cover letter. Guidelines for resubmitting your figure files are available below the reviewer comments at the end of this letter.

If applicable, we recommend that you deposit your laboratory protocols in protocols.io to enhance the reproducibility of your results. Protocols.io assigns your protocol its own identifier (DOI) so that it can be cited independently in the future. For instructions see: https://journals.plos.org/plosone/s/submission-guidelines#loc-laboratory-protocols. Additionally, PLOS ONE offers an option for publishing peer-reviewed Lab Protocol articles, which describe protocols hosted on protocols.io. Read more information on sharing protocols at https://plos.org/protocols?utm_medium=editorial-email&utm_source=authorletters&utm_campaign=protocols.

As the corresponding author, your ORCID iD is verified in the submission system and will appear in the published article. PLOS supports the use of ORCID, and we encourage all coauthors to register for an ORCID iD and use it as well. Please encourage your coauthors to verify their ORCID iD within the submission system before final acceptance, as unverified ORCID iDs will not appear in the published article. *Only*  the individual author can complete the verification step; PLOS staff *cannot*  verify ORCID iDs on behalf of authors.

We look forward to receiving your revised manuscript.

Kind regards,

Chunyu Zhang

Academic Editor

PLOS One

Journal Requirements:

If the reviewer comments include a recommendation to cite specific previously published works, please review and evaluate these publications to determine whether they are relevant and should be cited. There is no requirement to cite these works unless the editor has indicated otherwise.

**Additional Editor Comments:**

I encourage the authors to further clarify the boundary between occupational burnout as a psychological condition and burnout-related discourse as expressed in social media posts. The current manuscript sometimes moves too quickly from analysing online texts to making claims about workers’ actual burnout experiences, regional burnout levels, and collective emotional mobilisation. The authors should therefore consistently frame the findings as patterns observed in the analysed corpus of digitally active social media users, rather than as evidence about Chinese workers in general. Relatedly, likes should be treated more cautiously as a proxy for online engagement or resonance, not as direct evidence of collective identification, because they may also be shaped by platform algorithms, posting time, account visibility, text length, and user demographics. The spatial analysis also needs more caution, particularly where provincial sample sizes are small; otherwise, tertile-based sentiment categories may create an impression of regional burnout differences that the data cannot support. Finally, the manuscript would benefit from reducing rhetorically strong expressions such as collective mobilisation, emotional sovereignty, and high-conductivity material, and replacing them with more empirically grounded and methodologically neutral language. These revisions would help align the theoretical claims more closely with the exploratory, cross-sectional, and platform-mediated nature of the data.

[Note: HTML markup is below. Please do not edit.]

Reviewers' comments:

Reviewer's Responses to Questions

**Comments to the Author**

1. If the authors have adequately addressed your comments raised in a previous round of review and you feel that this manuscript is now acceptable for publication, you may indicate that here to bypass the “Comments to the Author” section, enter your conflict of interest statement in the “Confidential to Editor” section, and submit your "Accept" recommendation.

Reviewer #1: All comments have been addressed

Reviewer #4: (No Response)

**********

2. Is the manuscript technically sound, and do the data support the conclusions?

The manuscript must describe a technically sound piece of scientific research with data that supports the conclusions. Experiments must have been conducted rigorously, with appropriate controls, replication, and sample sizes. The conclusions must be drawn appropriately based on the data presented.

Reviewer #1: Yes

Reviewer #4: No

**********

3. Has the statistical analysis been performed appropriately and rigorously?

Reviewer #1: I Don't Know

Reviewer #4: No

**********

4. Have the authors made all data underlying the findings in their manuscript fully available?

The PLOS Data policy requires authors to make all data underlying the findings described in their manuscript fully available without restriction, with rare exception (please refer to the Data Availability Statement in the manuscript PDF file). The data should be provided as part of the manuscript or its supporting information, or deposited to a public repository. For example, in addition to summary statistics, the data points behind means, medians and variance measures should be available. If there are restrictions on publicly sharing data—e.g. participant privacy or use of data from a third party—those must be specified.

Reviewer #1: Yes

Reviewer #4: No

**********

5. Is the manuscript presented in an intelligible fashion and written in standard English?

PLOS ONE does not copyedit accepted manuscripts, so the language in submitted articles must be clear, correct, and unambiguous. Any typographical or grammatical errors should be corrected at revision, so please note any specific errors here.

Reviewer #1: Yes

Reviewer #4: Yes

**********

6. Review Comments to the Author

Please use the space provided to explain your answers to the questions above. You may also include additional comments for the author, including concerns about dual publication, research ethics, or publication ethics. (Please upload your review as an attachment if it exceeds 20,000 characters)

Reviewer #1: We would like to thank the authors and commend their efforts in addressing our comments and improving the submitted manuscript. However, there are still some aspects that could be clarified and improved, such as the following:

1) In the abstract: we suggest including the background, objective, methods, results and conclusion. Furthermore, the wording of the objectives should be improved; they should be stated as a purpose using non-finite verbs rather than as questions. In the methods section, briefly describe the type, design, sample, research techniques and instruments, and data analysis.

2) It is noted that the introduction has been improved; however, it is suggested that the logical coherence be reviewed and that the following aspects be included: a) General context of the problem, b) Digital transformation and social media as a data source, c) Theoretical and conceptual approaches, d) Computational approach, e) State of the art, f) Knowledge gap, g) Contribution and objectives of the study.

3) Regarding the methods section, the authors state that the observations were addressed and mention that “Regarding the type and design of the research, we have added a clear statement at the beginning of Section 3.1 of the revised manuscript, which defines this study as a computational social science investigation based on digital tracking data from social networks, with a cross-sectional, descriptive, and exploratory design. The research questions focus on the descriptive exploration and theoretical interpretation of the three dimensions of 'discourse, sentiment, and resonance,' rather than causal inference….” However, in the attached manuscript, Section 3, Line 272, 3.2, Research Design, this is not observed. It is suggested that the relevant changes be included in the new manuscript. The data processing and analysis should be described clearly and precisely.

4) The results should be presented in accordance with the research objectives. The study objectives can be inferred from the proposed problems.

5) Figures should not be included in the discussion; they should be included in the results section. Furthermore, it is suggested that the discussion interpret the results with critical analysis, considering the theoretical and conceptual framework and comparing them with previous studies. The theoretical and practical implications, as well as limitations, were also considered to enhance the discussion.

6) Regarding the conclusions, it is suggested that these be summarised in response to the research objectives, contributions, implications and scope.

Reviewer #4: Brief Summary

==========

The manuscript studies how workplace burnout is expressed on Chinese social media, using 3,691 texts from five platforms and combining BERTopic, Qwen-Plus assisted thematic coding, RoBERTa sentiment scoring, and regression modelling of likes as a proxy for resonance. It reports ten discourse themes, predominantly negative sentiment, regional variation in sentiment, and higher engagement for posts labelled as “institutional absurdity”. The core contribution is an integrated discourse-sentiment-resonance framework for analysing burnout as a social media phenomenon rather than only as an individual psychological construct. However, several claims about Chinese workers, regional burnout patterns, and affective mobilisation are stronger than the evidence supports, given the purposive sample, platform mediation, limited model validation, and under-controlled engagement analyses.

Major Comments

===========

#1. The manuscript repeatedly moves from a purposive corpus of digitally active social media users to claims about “Chinese workers”, “regions”, and “occupational burnout” more generally. Revise all general claims so that they refer to the analysed posts or users, and add per-platform and per-province sample counts with uncertainty indicators before retaining any spatial interpretation.

#2. The data collection and reproducibility description is not yet sufficiently auditable for PLOS ONE. Provide the exact crawling/API procedures, query dates, search strings, inclusion/exclusion counts before and after filtering, SimHash and bot-detection thresholds, code, model versions, package versions, random seeds, and resolve the inconsistency between the submission form marking ethics as “N/A” and the manuscript reporting full IRB approval.

#3. The topic pipeline depends on an exploratory BERTopic run, manual theme naming, and subsequent LLM classification, while the “Others” category still contains 35.38% of the corpus. Add a compact stability check across at least alternative seeds or clustering thresholds, report topic coherence or equivalent interpretability evidence, and provide a human-versus-LLM confusion matrix for the validation subset rather than only aggregate kappa.

#4. The sentiment analysis validation is incomplete because the manuscript reports targeted positive-class accuracy but not full performance over the sentiment classes. State the exact RoBERTa model, the mapping from classifier output to the [-1, 1] sentiment score, the class distribution, inter-annotator agreement, confusion matrix, macro-F1, and a sensitivity analysis showing whether the main conclusions survive exclusion or recoding of likely ironic cases.

#5. The resonance analysis over-interprets likes as collective identification while not controlling for major engagement confounders such as platform, time, text length, topic visibility, and possible account-level exposure. Re-estimate the ZINB and threshold models with the available platform and temporal covariates, report both the count and inflation components, and replace causal language such as “trigger” and “mobilisation” with explicitly associational wording unless stronger identification is provided.

#6. The related work should better position the computational sentiment component against recent studies on social media sentiment and metadata-aware modelling. For example, “Enhancing Sentiment Analysis on Social Media: Integrating Text and Metadata for Refined Insights” (2024) is a relevant reference point for explaining why this manuscript relies primarily on text and likes rather than richer metadata features.

Minor Comments

===========

#7. The title is inconsistent across the submission metadata and the manuscript body, where “Discourse, Sentiment, and Resonance of Workplace Burnout on Chinese Social Media: A Computational Analysis” and “What Burns You Out? Discourse, Sentiment and Resonance in Chinese Workplace” both appear. Use one final title consistently throughout the submission files.

#8. Several expressions require correction before publication, including “means wordking”, inconsistent capitalisation of RoBERTa, and over-definitive labels such as “low occupational burnout” for tertile-based sentiment categories. Replace these with grammatically correct and methodologically neutral wording.

#9. Figures and tables need more self-contained captions. For Figures 2–4 and Table 2, state the sample size, excluded observations, model specification, baseline category, covariates, and whether reported margins are derived from the ZINB count component.

**********

7. PLOS authors have the option to publish the peer review history of their article (what does this mean?). If published, this will include your full peer review and any attached files.

If you choose “no”, your identity will remain anonymous but your review may still be made public.

**Do you want your identity to be public for this peer review?**  For information about this choice, including consent withdrawal, please see our Privacy Policy.

Reviewer #1: No

Reviewer #4: No

**********

[NOTE: If reviewer comments were submitted as an attachment file, they will be attached to this email and accessible via the submission site. Please log into your account, locate the manuscript record, and check for the action link "View Attachments". If this link does not appear, there are no attachment files.]

To ensure your figures meet our technical requirements, please review our figure guidelines: https://journals.plos.org/plosone/s/figures

You may also use PLOS’s free figure tool, NAAS, to help you prepare publication quality figures: https://journals.plos.org/plosone/s/figures#loc-tools-for-figure-preparation.

NAAS will assess whether your figures meet our technical requirements by comparing each figure against our figure specifications.

# Author comment: Author response to Decision Letter 2

**DOI:** 10.1371/journal.pone.0346396.r004

14 Jun 2026

We thank the reviewer for the valuable guidance. Point-by-point responses are provided in the response letter.

Attachment Submitted filename: response-r2.doc

# Peer review: Decision Letter 2

**DOI:** 10.1371/journal.pone.0346396.r005

**Authors:** Chunyu Zhang

8 Jul 2026

PONE-D-26-05195R2

Discourse, Sentiment, and Resonance of Workplace Burnout on Chinese Social Media: A Computational Analysis

PLOS One

Dear Dr. Yin,

Thank you for submitting your manuscript to PLOS ONE. After careful consideration, we feel that it has merit but does not fully meet PLOS ONE’s publication criteria as it currently stands. Therefore, we invite you to submit a revised version of the manuscript that addresses the points raised during the review process.

Please submit your revised manuscript by Aug 22 2026 11:59PM. If you will need more time than this to complete your revisions, please reply to this message or contact the journal office at plosone@plos.org. When you're ready to submit your revision, log on to https://www.editorialmanager.com/pone/ and select the 'Submissions Needing Revision' folder to locate your manuscript file.

Please include the following items when submitting your revised manuscript:

- A letter that responds to each point raised by the academic editor and reviewer(s). You should upload this letter as a separate file labeled 'Response to Reviewers'.
- A marked-up copy of your manuscript that highlights changes made to the original version. You should upload this as a separate file labeled 'Revised Manuscript with Track Changes'.
- An unmarked version of your revised paper without tracked changes. You should upload this as a separate file labeled 'Manuscript'.

If you would like to make changes to your financial disclosure, please include your updated statement in your cover letter. Guidelines for resubmitting your figure files are available below the reviewer comments at the end of this letter.

If applicable, we recommend that you deposit your laboratory protocols in protocols.io to enhance the reproducibility of your results. Protocols.io assigns your protocol its own identifier (DOI) so that it can be cited independently in the future. For instructions see: https://journals.plos.org/plosone/s/submission-guidelines#loc-laboratory-protocols. Additionally, PLOS ONE offers an option for publishing peer-reviewed Lab Protocol articles, which describe protocols hosted on protocols.io. Read more information on sharing protocols at https://plos.org/protocols?utm_medium=editorial-email&utm_source=authorletters&utm_campaign=protocols.

As the corresponding author, your ORCID iD is verified in the submission system and will appear in the published article. PLOS supports the use of ORCID, and we encourage all coauthors to register for an ORCID iD and use it as well. Please encourage your coauthors to verify their ORCID iD within the submission system before final acceptance, as unverified ORCID iDs will not appear in the published article. *Only*  the individual author can complete the verification step; PLOS staff *cannot*  verify ORCID iDs on behalf of authors.

We look forward to receiving your revised manuscript.

Kind regards,

Chunyu Zhang

Academic Editor

PLOS One

Journal Requirements:

1. If the reviewer comments include a recommendation to cite specific previously published works, please review and evaluate these publications to determine whether they are relevant and should be cited. There is no requirement to cite these works unless the editor has indicated otherwise.

2. Please review your reference list to ensure that it is complete and correct. If you have cited papers that have been retracted, please include the rationale for doing so in the manuscript text, or remove these references and replace them with relevant current references. Any changes to the reference list should be mentioned in the rebuttal letter that accompanies your revised manuscript. If you need to cite a retracted article, indicate the article’s retracted status in the References list and also include a citation and full reference for the retraction notice.

Additional Editor Comments:

Thank you for submitting the revised manuscript. The reviewers agree that the manuscript has improved substantially, and one reviewer now recommends acceptance. I am also satisfied that the main methodological and presentation concerns raised in the previous round have largely been addressed. However, before the manuscript can be accepted, please complete a final minor revision to improve methodological transparency and presentation consistency. In particular, please clarify the sampling strategy and the selection process for the final 1,276 texts, including inclusion and exclusion criteria, scraping procedures, treatment of missing data or excluded cases, and the role of platform or account-level controls. Please also define OLS and log regression clearly, provide a brief explanation of the OLS model, justify the use of PCA, and report the robustness analyses more explicitly. Please ensure that the research design is described consistently throughout the manuscript, that all acronyms and methodological terms are defined at first use, and that the Data Availability Statement and OSF repository provide sufficient materials for verification and replication. Finally, please submit a clean version of the manuscript free of tracked changes, duplicated text, formatting problems, or residual text from earlier revisions. Please provide a point-by-point response explaining how each of these remaining issues has been addressed.

[Note: HTML markup is below. Please do not edit.]

Reviewer's Responses to Questions

**Comments to the Author**

1. If the authors have adequately addressed your comments raised in a previous round of review and you feel that this manuscript is now acceptable for publication, you may indicate that here to bypass the “Comments to the Author” section, enter your conflict of interest statement in the “Confidential to Editor” section, and submit your "Accept" recommendation.

Reviewer #1: All comments have been addressed

Reviewer #4: All comments have been addressed

**********

2. Is the manuscript technically sound, and do the data support the conclusions?

The manuscript must describe a technically sound piece of scientific research with data that supports the conclusions. Experiments must have been conducted rigorously, with appropriate controls, replication, and sample sizes. The conclusions must be drawn appropriately based on the data presented.

Reviewer #1: Yes

Reviewer #4: Yes

**********

3. Has the statistical analysis been performed appropriately and rigorously?

Reviewer #1: Yes

Reviewer #4: Yes

**********

4. Have the authors made all data underlying the findings in their manuscript fully available?

The PLOS Data policy requires authors to make all data underlying the findings described in their manuscript fully available without restriction, with rare exception (please refer to the Data Availability Statement in the manuscript PDF file). The data should be provided as part of the manuscript or its supporting information, or deposited to a public repository. For example, in addition to summary statistics, the data points behind means, medians and variance measures should be available. If there are restrictions on publicly sharing data—e.g. participant privacy or use of data from a third party—those must be specified.

Reviewer #1: Yes

Reviewer #4: Yes

**********

5. Is the manuscript presented in an intelligible fashion and written in standard English?

PLOS ONE does not copyedit accepted manuscripts, so the language in submitted articles must be clear, correct, and unambiguous. Any typographical or grammatical errors should be corrected at revision, so please note any specific errors here.

Reviewer #1: Yes

Reviewer #4: Yes

**********

6. Review Comments to the Author

Please use the space provided to explain your answers to the questions above. You may also include additional comments for the author, including concerns about dual publication, research ethics, or publication ethics. (Please upload your review as an attachment if it exceeds 20,000 characters)

Reviewer #1: We would like to thank and congratulate the authors for their effort and dedication in addressing the comments on the revised manuscript. The manuscript has been reviewed and found to be methodologically sound, with adequate logical consistency. The statistical procedures are, in general, appropriate and consistent with the stated objectives. However, it is recommended that methodological transparency be enhanced by describing the sampling strategy, clarifying the meaning of the acronyms ‘OLS’ and ‘log regression’, and providing a brief description of the OLS model; justify the use of PCA; justify the final sample size of 1,276 texts (how they were selected, inclusion criteria, scraping strategies); report the robustness analysis; mention how missing data or exclusions were handled; and mention whether there were any other important control variables. Tone down some interpretative statements to avoid causal inferences that exceed the scope of a cross-sectional design, as this could lead to the risk of over-interpretation. Reconsider whether the methodological design should be an ‘observational computational social science study’ or a ‘cross-sectional observational computational social science study’. The widely recognised research paradigms are: positivist, post-positivist, constructivist, critical and pragmatic.

Reviewer #4: The authors have addressed the main methodological and presentation concerns raised in the previous round. I therefore recommend acceptance.

**********

7. PLOS authors have the option to publish the peer review history of their article (what does this mean?). If published, this will include your full peer review and any attached files.

If you choose “no”, your identity will remain anonymous but your review may still be made public.

**Do you want your identity to be public for this peer review?**  For information about this choice, including consent withdrawal, please see our Privacy Policy.

Reviewer #1: No

Reviewer #4: No

**********

[NOTE: If reviewer comments were submitted as an attachment file, they will be attached to this email and accessible via the submission site. Please log into your account, locate the manuscript record, and check for the action link "View Attachments". If this link does not appear, there are no attachment files.]

To ensure your figures meet our technical requirements, please review our figure guidelines: https://journals.plos.org/plosone/s/figures

You may also use PLOS’s free figure tool, NAAS, to help you prepare publication quality figures: https://journals.plos.org/plosone/s/figures#loc-tools-for-figure-preparation.

NAAS will assess whether your figures meet our technical requirements by comparing each figure against our figure specifications.

# Author comment: Author response to Decision Letter 3

**DOI:** 10.1371/journal.pone.0346396.r006

11 Jul 2026

We thank the reviewers for their patient guidance. We have completed the revisions point by point, and the details can be found in the uploaded Response Letter.

Attachment Submitted filename: Response_to_Reviewers_R3.docx

# Editor report: Decision Letter 3

**DOI:** 10.1371/journal.pone.0346396.r007

**Authors:** Chunyu Zhang

22 Jul 2026

Discourse, Sentiment, and Resonance of Workplace Burnout on Chinese Social Media: A Computational Analysis

PONE-D-26-05195R3

Dear Dr. Yin,

We’re pleased to inform you that your manuscript has been judged scientifically suitable for publication and will be formally accepted for publication once it meets all outstanding technical requirements.

Within one week, you’ll receive an e-mail detailing the required amendments. When these have been addressed, you’ll receive a formal acceptance letter and your manuscript will be scheduled for publication.

An invoice will be generated when your article is formally accepted. Please note, if your institution has a publishing partnership with PLOS and your article meets the relevant criteria, all or part of your publication costs will be covered. Please make sure your user information is up-to-date by logging into Editorial Manager at Editorial Manager® and clicking the ‘Update My Information' link at the top of the page. For questions related to billing, please contact billing support.

If your institution or institutions have a press office, please notify them about your upcoming paper to help maximize its impact. If they’ll be preparing press materials, please inform our press team as soon as possible -- no later than 48 hours after receiving the formal acceptance. Your manuscript will remain under strict press embargo until 2 pm Eastern Time on the date of publication. For more information, please contact onepress@plos.org.

Kind regards,

Chunyu Zhang

Academic Editor

PLOS One

Additional Editor Comments (optional):

Reviewers' comments:

# Editor report: Acceptance letter

**DOI:** 10.1371/journal.pone.0346396.r008

**Authors:** Chunyu Zhang

PONE-D-26-05195R3

PLOS One

Dear Dr. Yin,

I'm pleased to inform you that your manuscript has been deemed suitable for publication in PLOS One. Congratulations! Your manuscript is now being handed over to our production team.

At this stage, our production department will prepare your paper for publication. This includes ensuring the following:

* All references, tables, and figures are properly cited

* All relevant supporting information is included in the manuscript submission,

* There are no issues that prevent the paper from being properly typeset

You will receive further instructions from the production team, including instructions on how to review your proof when it is ready. Please keep in mind that we are working through a large volume of accepted articles, so please give us a few days to review your paper and let you know the next and final steps.

Lastly, if your institution or institutions have a press office, please let them know about your upcoming paper now to help maximize its impact. If they'll be preparing press materials, please inform our press team within the next 48 hours. Your manuscript will remain under strict press embargo until 2 pm Eastern Time on the date of publication. For more information, please contact onepress@plos.org.

You will receive an invoice from PLOS for your publication fee after your manuscript has reached the completed accept phase. If you receive an email requesting payment before acceptance or for any other service, this may be a phishing scheme. Learn how to identify phishing emails and protect your accounts at https://explore.plos.org/phishing.

If we can help with anything else, please email us at customercare@plos.org.

Thank you for submitting your work to PLOS ONE and supporting open access.

Kind regards,

PLOS ONE Editorial Office Staff

on behalf of

Dr. Chunyu Zhang

Academic Editor

PLOS One
